# In Vitro Metabolic Fate of the Synthetic Cannabinoid Receptor Agonists QMPSB and QMPCB (SGT-11) Including Isozyme Mapping and Esterase Activity

**DOI:** 10.3390/metabo11080509

**Published:** 2021-08-03

**Authors:** Matthias J. Richter, Lea Wagmann, Tanja M. Gampfer, Simon D. Brandt, Markus R. Meyer

**Affiliations:** 1 Department of Experimental and Clinical Toxicology, Institute of Experimental and Clinical Pharmacologyand Toxicology, Center for Molecular Signaling (PZMS), Saarland University, 66421 Homburg, Germany; Matthias.Richter@uks.eu (M.J.R.); Lea.Wagmann@uks.eu (L.W.); Tanja.Gampfer@uks.eu (T.M.G.); 2 School of Pharmacy and Biomolecular Sciences, Liverpool John Moores University, Liverpool L3 3AF, UK; s.brandt@ljmu.ac.uk

**Keywords:** QMPSB, QMPCB, synthetic cannabinoid receptor agonists, SCRA, NPS, metabolism, LC-HRMS/MS

## Abstract

Quinolin-8-yl 4-methyl-3-(piperidine-1-sulfonyl)benzoate (QMPSB) and quinolin-8-yl 4-methyl-3-(piperidine-1-carbonyl)benzoate (QMPCB, SGT-11) are synthetic cannabinoid receptor agonists (SCRAs). Knowing their metabolic fate is crucial for the identification of toxicological screening targets and to predict possible drug interactions. The presented study aimed to identify the in vitro phase I/II metabolites of QMPSB and QMPCB and to study the contribution of different monooxygenases and human carboxylesterases by using pooled human liver S9 fraction (pHLS9), recombinant human monooxygenases, three recombinant human carboxylesterases, and pooled human liver microsomes. Analyses were carried out by liquid chromatography high-resolution tandem mass spectrometry. QMPSB and QMPCB showed ester hydrolysis, and hydroxy and carboxylic acid products were detected in both cases. Mono/dihydroxy metabolites were formed, as were corresponding glucuronides and sulfates. Most of the metabolites could be detected in positive ionization mode with the exception of some QMPSB metabolites, which could only be found in negative mode. Monooxygenase activity screening revealed that CYP2B6/CYP2C8/CYP2C9/CYP2C19/CYP3A4/CYP3A5 were involved in hydroxylations. Esterase screening showed the involvement of all investigated isoforms. Additionally, extensive non-enzymatic ester hydrolysis was observed. Considering the results of the in vitro experiments, inclusion of the ester hydrolysis products and their glucuronides and monohydroxy metabolites into toxicological screening procedures is recommended.

## 1. Introduction

Synthetic cannabinoids, also known as synthetic cannabinoid receptor agonists (SCRAs), represent one of the largest groups of the so-called new psychoactive substances (NPSs) [1,2]. SCRAs are often sold on the drug market after being sprayed on plant material [3]. Continuous changes in the chemical structures lead to a continuous introduction of new SCRAs in an attempt to circumvent legislation [3]. Even though the number of new SCRAs detected annually in the EU decreased in recent years, they continue to represent an important group of newly occurring NPSs alongside cathinones and opioids [2].

**Figure 1:**
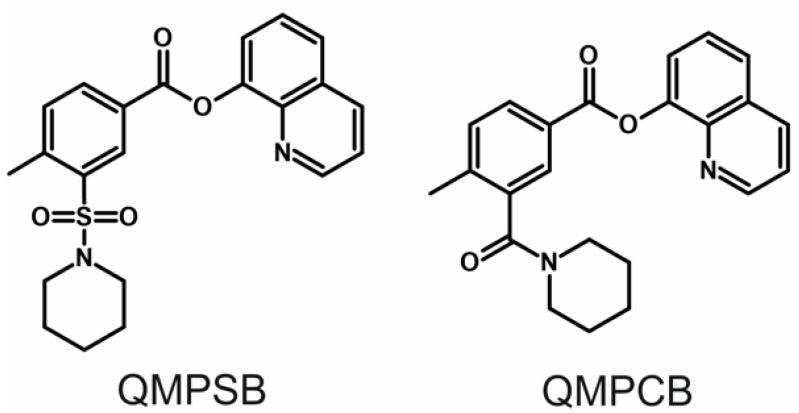
Chemical structures of the studied compounds.

In 2007, quinolin-8-yl 4-methyl-3-(piperidine-1-sulfonyl)benzoate (QMPSB) was identified as a potent SCRA as a result of a high-throughput screening [4]. Further investigations revealed that QMPSB is a potent full agonist at the CB1 and CB2 receptors with moderate selectivity for the CB2 receptor [4,5]. In Queensland (Australia), QMPSB was found in a large number of plant materials seized in 2011 and 2012 and published in 2016 [6]. QMPSB was also the template for a new group of SCRAs based on a sulfamoyl benzoate structure and/or a quinolin-8-yl ester head group. In 2012, the company Stargate International started research on this new SCRA group which included the amide analog of QMPSB, quinolin-8-yl 4-methyl-3-(piperidine-1-carbonyl)benzoate (QMPCB, SGT-11). Chemical structures of QMPSB and QMPCB can be found in Figure 1. By replacing the sulfamoyl tail group linker with a carbonyl linker, the influence of the linker group on the pharmacological properties was further investigated. However, tests on human volunteers showed that the amide analog QMPCB had a lower potency than QMPSB [7]. Further structural modifications led to a combination of the quinolin-8-yl ester head group with an *N*-alkyl-1*H*-indole core, which resulted in the development and appearance of PB-22 (QUPIC), and BB-22 (QUCHIC) amongst others [7,8].

In the context of clinical and forensic toxicology, data on the metabolism of emerging NPSs are important, particularly for developing analytical screening procedures in urine. To our knowledge, no data on the metabolism of QMPSB and QMPCB are available in the literature. Therefore, the aim of the study was to identify the metabolites of these substances as potential targets for toxicological screenings by liquid chromatography coupled to high-resolution tandem mass spectrometry (LC-HRMS/MS). In vitro incubations using pooled human liver S9 fraction (pHLS9) were performed to identify phase I and II metabolites. Incubations with pHLS9 have been shown to generate the main human metabolites compared to human primary hepatocytes and are therefore appropriate alternatives to incubations with human primary hepatocytes [9].

An in vitro monooxygenase activity assay should clarify the involvement of individual monooxygenases in the initial metabolic steps. Due to the instability of the ester bond already described in the literature [5,6], the additional influence of human esterases on ester hydrolysis was investigated.

## 2. Results and Discussion

### 2.1. Identification of In Vitro Phase I and II Metabolites of QMPSB

For metabolite identification, exact protonated or deprotonated precursor ion (PI) masses of expected phase I and II metabolites were calculated. High-resolution full scan data were examined for these masses and the corresponding MS^2^ spectra were interpreted. Deviations between measured and calculated PI mass were only accepted up to 5 ppm. All metabolites were tentatively identified since no reference material was available.

In total, 17 phase I and 4 phase II QMPSB metabolites were identified in pHLS9 or CYP isozyme incubations (metabolites SM1–SM21; Table S1 in the Electronic Supplementary Material, ESM). The ionization mode (positive or negative) and the incubation type (pHLS9 or CYP isozyme incubation) in which the respective metabolite was found are listed in Table S1 (ESM).

**Figure 2:**
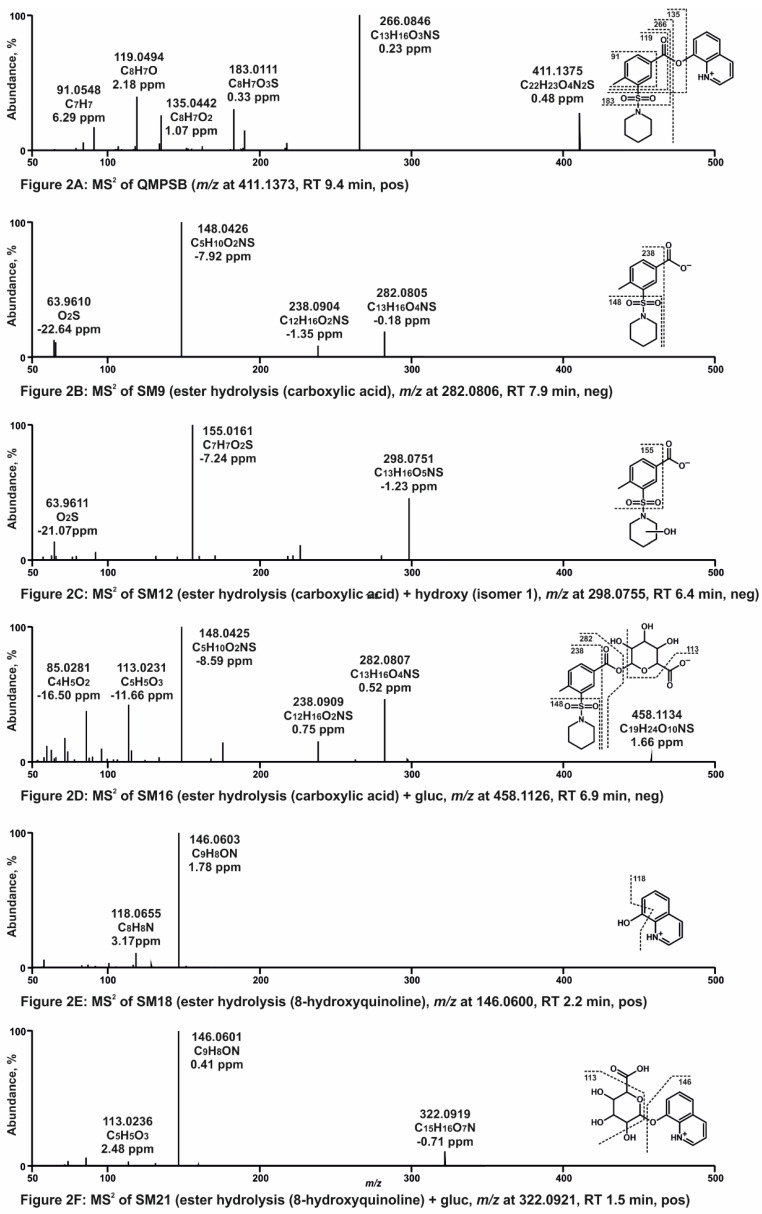
(**A**–**F**) High-resolution MS^2^ spectra of QMPSB and most abundant QMPSB metabolites detected in pooled human liver S9 incubations (SM9, SM12, SM16, SM18, and SM21). RT, retention time; pos, positive ionization mode; neg, negative ionization mode; gluc, glucuronic acid.

Nine metabolites were identified in pHLS9 incubations, which were ester hydrolysis products and metabolites derived therefrom. No metabolite with an intact ester moiety was found. It is important to mention that the ester hydrolysis products (SM9 and SM18 in Table S1) were also detectable in negative control samples without pHLS9 with an abundant signal. This indicates that a rapid non-enzymatic ester hydrolysis took place. The MS^2^ spectra of QMPSB and most abundant metabolites of pHLS9 incubation are given in Figure 2A–F. Chromatograms of these compounds are shown in Figure S1 (ESM). MS^2^ spectra of further metabolites can be found in Figure S3 (ESM). The obtained MS^2^ spectrum of QMPSB (Figure 2A) is comparable with data reported previously [6,7]. The base peak is formed by the benzoyl ion with *m/z* 266.0845 (C_13_H_16_O_3_NS) after ester cleavage. Further loss of the piperidine ring leads to fragment ion (FI) at *m/z* 183.0110 (C_8_H_7_O_3_S), followed by loss of SO_2_ and CO, represented by the FIs at *m/z* 119.0491 (C_8_H_7_O) and *m/z* 91.0542 (C_7_H_7_). Ester hydrolysis forms metabolite SM9 (Figure 2B) with protonated PI at *m/z* 282.0806 (C_13_H_16_O_4_NS). Loss of CO_2_ and 4-methyl benzoate resulted in the FIs at *m/z* 238.0907 (C_12_H_16_O_2_NS) and *m/z* 148.0438 (C_5_H_10_O_2_NS). SM12 (Figure 2C) with PI at *m/z* 298.0755 (C_13_H_16_O_5_NS) appears to be hydroxylated at the piperidine ring which is indicated by the FI at *m/z* 155.0172 (C_7_H_7_O_2_S) obtained after the loss of CO_2_ and the hydroxylated piperidine ring. Metabolite SM16 (Figure 2D) with PI at *m/z* 458.1126 (C_19_H_24_O_10_NS) is the glucuronidated ester hydrolysis product indicated by the two most abundant FIs at *m/z* 282.0806 (C_13_H_16_O_4_NS) and *m/z* 148.0438 (C_5_H_10_O_2_NS), which were the most abundant signals in the spectrum of the non-glucuronidated metabolite SM9. The ester hydrolysis product 8-hydroxyquinoline (SM18 in Figure 2E) with PI at *m/z* 146.0600 (C_9_H_8_ON) shows a loss of CO resulting in the FI at *m*/*z* 118.0651 (C_8_H_8_N). Metabolite SM21 (Figure 2F), the glucuronidated 8-hydroxyquinoline, is characterized by FI at *m*/*z* 146.0600 (C_9_H_8_ON), which corresponds to the PI of SM18 in Figure 2E. The MS^2^ spectra of further metabolites are given in Figure S3 (ESM).

Due to the expected ester hydrolysis products with carboxylic acid function, HRMS/MS analysis was also carried out in negative ionization mode. In fact, the majority of metabolites derived from the carboxylic acid part after ester hydrolysis (SM10, SM11, SM12, SM13, SM14, SM15, and SM16 in Table S1) were only detected with negative ionization. Therefore, the use of negative ionization mode in addition to positive ionization mode can increase the detectability of QMPSB metabolites and should be recommended when developing screening strategies.

### 2.2. Identification of In Vitro Phase I and II Metabolites of QMPCB

In total, 30 phase I and 4 phase II metabolites of QMPCB were tentatively identified in pHLS9 or CYP isozyme incubations (CM1–CM34). The ionization mode (positive or negative) and the incubation type (pHLS9 or CYP isozyme incubation) in which the respective metabolite was found are listed in Table S2 (ESM).

**Figure 3:**
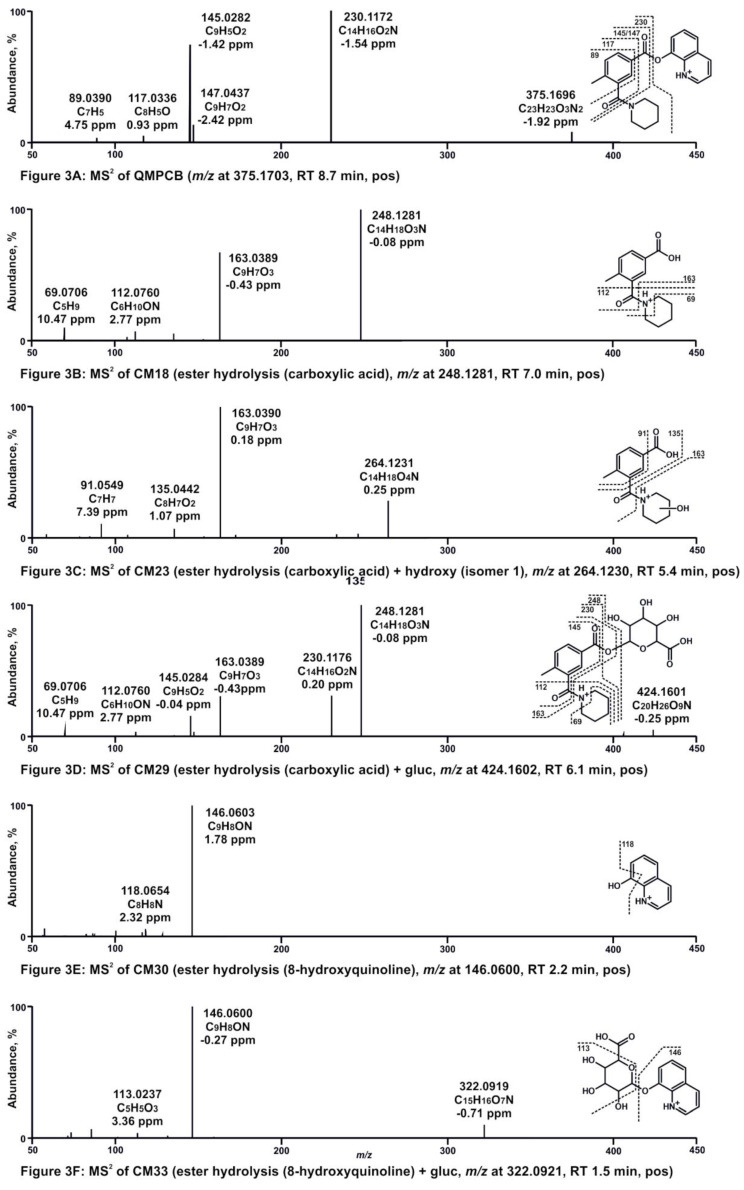
(**A**–**F**) High-resolution MS^2^ spectra of QMPCB and most abundant QMPCB metabolites detected in pooled human liver S9 incubations (CM18, CM23, CM29, CM30, and CM33). RT, retention time; pos, positive ionization mode; neg, negative ionization mode; gluc, glucuronic acid.

Nine metabolites were identified in pHLS9 incubations. Similar to the pHLS9 incubation results of QMPSB, all metabolites were ester hydrolysis products and metabolites derived therefrom. Ester hydrolysis products were also found in control samples without pHLS9 with an abundant signal, which indicated that non-enzymatic hydrolysis of QMPCB occurred, comparable to QMPSB. The MS^2^ spectra of QMPCB and most abundant metabolites formed in pHLS9 incubation are depicted in Figure 3A–F. Chromatograms of these compounds are shown in Figure S2 (ESM). MS^2^ spectra of additional metabolites can be found in Figure S4 (ESM). The obtained MS^2^ spectrum of QMPCB (Figure 3A) is comparable with data reported in a previous publication [7]. As with QMPSB, the base peak is formed by the benzoyl ion at *m/z* 230.1176 (C_14_H_16_O_2_N) after ester cleavage. Further cleavage of the amide bond leads to the FI at *m/z* 145.0284 (C_9_H_5_O_2_), followed by two losses of CO, represented by the FIs at *m/z* 117.0335 (C_8_H_5_O) and *m/z* 89.0386 (C_7_H_5_). Ester hydrolysis forms metabolite CM18 (Figure 3B) with protonated PI at *m/z* 248.1281 (C_14_H_18_O_3_N). Loss of piperidine resulting in FI at *m/z* 163.0390 (C_9_H_7_O_3_). FIs at *m/z* 69.0699 (C_5_H_9_) and *m/z* 112.0757 (C_6_H_10_ON) are caused by the alkyl chain of piperidine with and without amide group. Metabolite CM23 (Figure 3C) with PI at *m/z* 264.1230 (C_14_H_18_O_4_N) appears to be hydroxylated at the piperidine ring, which is indicated by the FI at *m/z* 163.0390 (C_9_H_7_O_3_). Metabolite CM29 (Figure 3D) with PI at *m/z* 424.1602 (C_20_H_26_O_9_N) is the glucuronide of metabolite CM18, resulting in the FIs at *m/z* 248.1281 (C_14_H_18_O_3_N) and *m/z* 163.0390 (C_9_H_7_O_3_), which are also the most abundant fragments of CM18. The ester hydrolysis product 8-hydroxyquinoline was found as metabolite CM30 (Figure 3E). CM30 and the derived glucuronide (CM33 in Figure 3F) are also metabolites of QMPSB (SM18 and SM21) and have already been described there. MS^2^ spectra data of further metabolites are given in Figure S4 (ESM).

Almost all QMPCB metabolites were detected in positive ionization mode (Table S2). CM27 and CM28, two dihydroxy metabolites of the carboxylic acid product after ester hydrolysis, were only detectable in negative ionization mode. Improvement in detectability of the metabolites due to the additional negative ionization mode seemed to be of minor importance in the case of QMPCB compared to QMPSB.

### 2.3. Proposed Metabolic Pathways of QMPSB

**Figure 4:**
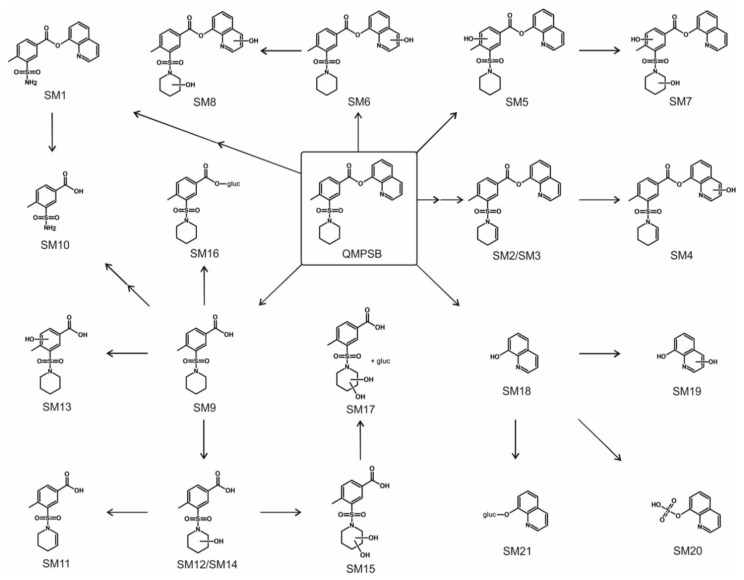
Metabolic pathways of QMPSB. SM1–SM21, metabolites of QMPSB; gluc, glucuronic acid.

The proposed metabolic pathways are shown in Figure 4. For in vitro metabolism of QMPSB, ester hydrolysis has been revealed to be an important step. Therefore, the identified metabolites were sorted into three groups: metabolites with intact ester group (SM1–SM8 in Figure 4), and the two ester hydrolysis products with additional metabolic reactions: carboxylic acid part (SM9–SM17 in Figure 4) and 8-hydroxyquinoline (SM18–SM21 in Figure 4). All metabolites with an intact ester group (SM1–SM8 in Figure 4), as well as SM10, SM13, SM14, and SM19 (in Figure 4), were only identified in CYP incubations, not in pHLS9 incubation. The reason for these findings could be that the phase I metabolites formed during the pHLS9 incubation were further metabolized to phase II metabolites, whereby the formed phase I metabolites were below the detection limit. In addition, compared to CYP incubations, human carboxylesterases were present in pHLS9 incubations and the incubation time was longer in pHLS9 incubations, which could have contributed to increased ester hydrolysis. Therefore, pHLS9 metabolites are expected to have more pronounced in vivo relevance than the metabolites that were additionally detected in the recombinant CYP isozyme incubations. For the SCRA FUB-PB-22, which also contains a quinolin-8-yl ester head group but an *N*-alkyl-1H-indole core, metabolites found in authentic urine samples were described in the literature, and neither the parent drug nor metabolites with intact ester were found [10].

Starting from the carboxylic acid after ester hydrolysis (SM9 in Figure 4), three monohydroxy metabolites could be found: SM13, which is hydroxylated at the 4-methylbenzoate core, and SM12 and SM14 (Figure 4), which are hydroxylated at the piperidine ring. The exact position of the hydroxy group on the core or piperidine cannot be determined from the fragmentation. After the elimination of water, the dehydro metabolite SM11 (Figure 4) was formed with a double bond at the piperidine ring (the exact position in the ring is unknown). It should also be noted that a potential hydroxylation at the α-position to the nitrogen atom of the piperidine ring could lead to ring-opening. This mechanism was already described in the literature for other substances [11,12]. Further metabolic steps, however, can lead to cleavage of the C5 alkyl chain of piperidine, which could be detected with the metabolite SM10 (Figure 4). A dihydroxy metabolite was also found (SM15). In phase II metabolism of the carboxylic acid part, the glucuronides of the metabolites SM9 and SM15 could be detected: SM16 and SM17 (Figure 4).

8-Hydroxyquinoline (SM18 in Figure 4) was found to be hydroxylated (SM19 in Figure 4), although the exact position of the hydroxy group cannot be identified. In phase II metabolism, sulfation (SM20 in Figure 4) and glucuronidation (SM21 in Figure 4) of 8-hydroxyquinoline could be detected. The metabolites SM19 and SM20 have already been described in previous studies involving related SCRAs [9,10].

In pHLS9 incubations, the ester hydrolysis products SM9 and SM18 (Figure 4) as well as their glucuronides SM16 and SM21 (Figure 4) and the monohydroxy metabolite SM12 (Figure 4) showed the most abundant signals. Therefore, these metabolites are suggested as main targets for toxicological screenings. However, it is important to note that 8-hydroxyquinoline (SM18) and metabolites derived from it are not specific metabolites of QMPSB, but they can provide a hint for an SCRA with this partial structure.

### 2.4. Proposed Metabolic Pathways of QMPCB

**Figure 5:**
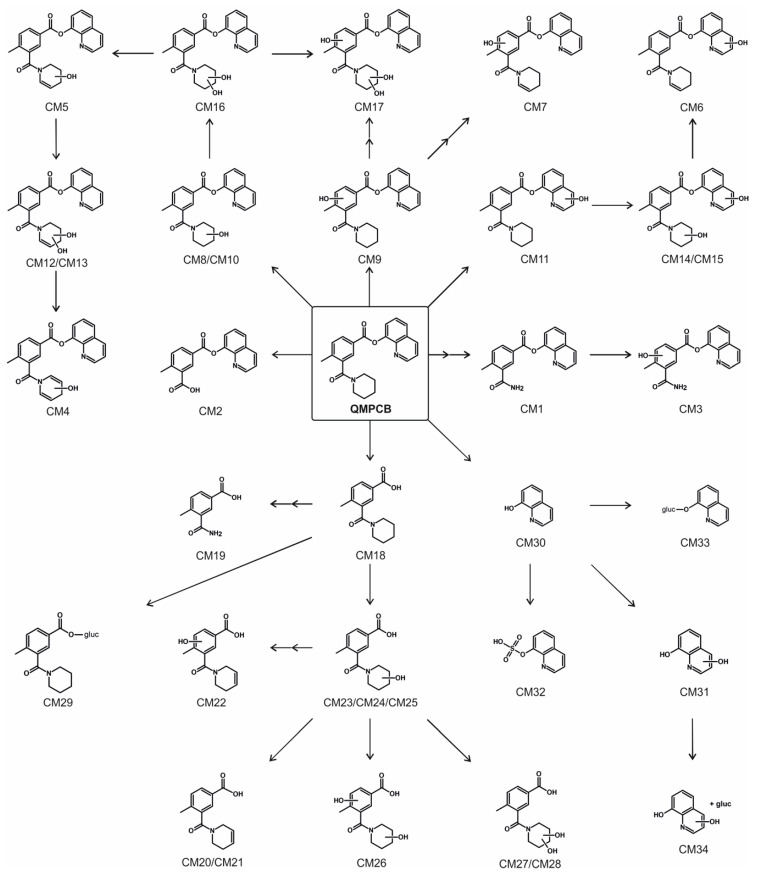
Metabolic pathways of QMPCB. CM1–CM34, metabolites of QMPCB; gluc, glucuronic acid.

The proposed metabolic pathways of QMPCB are depicted in Figure 5. During the in vitro metabolism of QMPCB, similar to QMPSB, ester hydrolysis played an important role. Therefore, the metabolites were also sorted into three groups: metabolites with an intact ester group (CM1–CM17 in Figure 5) and the two ester hydrolysis products with additional metabolic reactions: carboxylic acid part (CM18–CM29 in Figure 5) and 8-hydroxyquinoline (CM30–CM34 in Figure 5). Similar to QMPSB, all QMPCB metabolites with an intact ester group (CM1–CM17 in Figure 5) were only identified in CYP incubations. These findings confirm the instability of the ester bond with 8-hydroxyquinoline under pHLS9 incubation conditions. Some metabolites originating from ester hydrolysis could only be detected in CYP incubations: CM19–CM22, CM24, CM26, CM27, and CM31 (Figure 5). These metabolites are predominantly the result of a combination of different metabolic reactions, such as dihydroxylation or hydroxylation plus dehydrogenation. The reasons for these results are given in Section 2.3. MS^2^ spectra of these metabolites are given in Figure S4 (ESM).

Starting from the carboxylic acid part after ester hydrolysis, three monohydroxy metabolites could be found similar to QMPSB: CM23, CM24, and CM25 (Figure 5). In all three metabolites, the hydroxyl group is located on the piperidine ring. A ring-opening of the piperidine after hydroxylation at the α-position to the nitrogen atom of the piperidine ring [11,12] could also take place in the QMPCB, as already described above for QMPSB. The *N,N*-dealkyl metabolite could also be found (CM19 in Figure 5). In the case of the dihydroxy metabolites, the 4-methylbenzoate core and piperidine ring were hydroxylated (CM26 in Figure 5), or the piperidine ring was hydroxylated twice (CM27/CM28 in Figure 5). During phase II metabolism, the glucuronide of the carboxylic acid part following ester hydrolysis could be found analogous to QMPSB metabolism.

8-Hydroxyquinoline (CM30 in Figure 5) and the corresponding monohydroxy (CM31 in Figure 5), sulfate (CM32 in Figure 5), and glucuronide metabolites (CM33 in Figure 5) could also be found, which paralleled the observations made from QMPSB incubations. In addition, the glucuronide of the monohydroxy metabolite (CM34 in Figure 5) could be detected.

In pHLS9 incubations, the ester hydrolysis products CM18 and CM30 (Figure 5) as well as their glucuronides CM29 and CM33 (Figure 5) and two monohydroxy metabolites CM23 and CM25 (Figure 5) showed the most abundant signals. Therefore, these metabolites are suggested as main targets for toxicological screenings. As already mentioned with QMPSB, 8-hydroxyquinoline (CM30 in Figure 5) and metabolites derived from it are not specific metabolites of QMPCB, but they can provide an indication for a SCRA with this partial structure.

### 2.5. Monooxygenase Activity Screening of QMPSB and QMPCB

The monooxygenase activity screening revealed the involvement of several monooxygenases in the metabolism of QMPSB and QMPCB (Tables S3 and S4 in the ESM). CYP2B6, CYP2C8, CYP2C9, CYP2C19, CYP3A4, and CYP3A5 were involved in most metabolic reactions of both SCRAs. Although the quantitative involvement of each isoform was no investigated, it is quite unlikely that a drug-drug interaction causing the inhibition of a single CYP isozyme will lead to a significant change in hepatic clearance given that several isoforms were involved. The same can be assumed for poor metabolizers of single CYP isozymes.

### 2.6. Esterases Activity Screening with Recombinant hCES1b, hCES1c, hCES2, pHLM, and pHLS9

QMPSB and QMPCB both contain a quinolin-8-yl ester head group. According to previous reports, the chemical instability of this ester head group was already described in form of hydrolysis [5] and trans-esterification with solvents such as methanol and ethanol [6]. This must be considered when extracting them from seized plant material or human samples like plasma or urine. To avoid trans-esterification during storage, the stock solution for all experiments was therefore made with acetonitrile instead of methanol.

Different human carboxylesterase (hCES) isoforms catalyze the hydrolysis of ester-containing drugs [13,14]. The isoforms hCES1b and hCES1c are primarily expressed in the liver, and hCES2 is mainly expressed in the gastrointestinal tract [13]. In addition to the recombinant isoforms, the substrates were incubated with pHLM and pHLS9, which represent the natural hCES spectrum of the human liver. Negative control incubations (without enzyme) were conducted to evaluate non-enzymatic ester hydrolysis.

**Figure 6:**
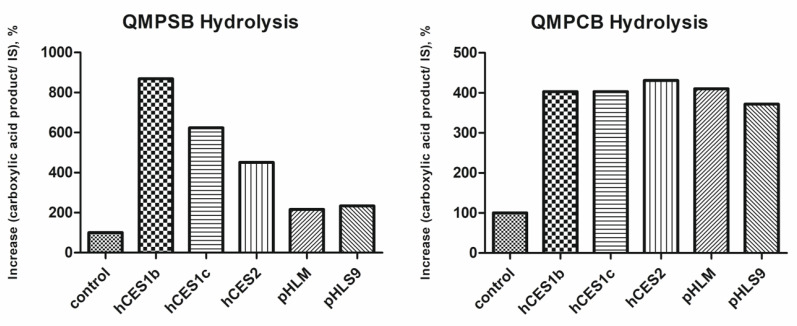
Esterases activity screening results: amount of formed hydrolysis product of QMPSB or QMPCB normalized to the internal standard (IS) and relative to the control incubation using different enzyme sources. Shown data represent mean of duplicate determination. hCES, human carboxylesterase; pHLM, pooled human liver microsomes; pHLS9, pooled human liver S9 fraction.

The ester hydrolysis product (carboxylic acid part) was measured to investigate the extent of ester hydrolysis. Therefore, the increase in the carboxylic acid product peak area normalized to the internal standard (trimipramine-d_3_) between 0 and 60 min of incubation time was determined. Results are shown in Figure 6. The control incubations of QMPSB and QMPCB show an increase in the ester hydrolysis products that must be the result of non-enzymatic ester hydrolysis. The increase in the carboxylic acid product of the control incubations was set to 100%, and all the other incubations were normalized (Figure 6).

Incubations with hCES1b, hCES1c, and hCES2 revealed a remarkable increase in ester hydrolysis rate of both SCRAs relative to non-enzymatic hydrolysis of the control incubations. For QMPSB, hCES1b showed the highest activity of all three isoforms. For QMPCB, there was no detectable difference between the three isoforms. This was mostly due to almost complete degradation of the parent molecule after 60 min incubation time. Incubations with pHLM and pHLS9 revealed an increase in ester hydrolysis rate of both SCRAs in comparison to the control incubations, but the increase was greater with QMPCB than with QMPSB. Since the carboxylesterase activities of pHLS9 and pHLM were not tested by the manufacturer, a quantitative comparison of their esterase activity with the isozymes was not possible, and the current incubations were primarily intended to provide qualitative evidence that the hydrolysis of the two SCRAs is catalyzed by the tested hCES isoforms and by the esterases contained in pHLM and pHLS9. The results of the human carboxylesterases are in agreement with a previous study on the related SCRAs BB-22 and PB-22, in which ester hydrolysis catalyzed by hCES1 and hCES2 could be demonstrated for both substances [15]. In this study, however, no non-enzymatic ester hydrolysis could be determined at physiological pH, which was possibly due to the shorter incubation time of 20 min [15].

These findings indicate that ester hydrolysis plays an important role in phase I metabolism of QMPSB and QMPCB. During sampling and handling, the non-enzymatic ester degradation should always be taken into account. To reduce substrate degradation, both SCRAs should be kept in solvents that cannot cause hydrolysis or transesterification.

## 3. Materials and Methods

### 3.1. Chemicals and Reagents

QMPSB and QMPCB were obtained from the library of Stargate International (Auckland, New Zealand) [7]. Stock solutions of QMPSB and QMPCB were prepared in acetonitrile (1 mg/mL) and stored at −18 °C. Trimipramine-d_3_ was obtained from LGC (Wesel, Germany). Isocitrate, isocitrate dehydrogenase, superoxide dismutase, 3′-phosphoadenosine-5′phosphosulfate (PAPS), S-(5′-adenosyl)-L-methionine (SAM), dithiothreitol, reduced glutathione, acetyl coenzyme A, magnesium chloride (MgCl_2_), potassium dihydrogen phosphate (KH_2_PO_4_), dipotassium hydrogen phosphate (K_2_HPO_4_), and Tris hydrochloride were obtained from Sigma-Aldrich (Taufkirchen, Germany). NADP^+^ was from Biomol (Hamburg, Germany). Baculovirus-infected insect cell microsomes (Supersomes) containing the human cDNA-expressed cytochrome P450 isozymes CYP1A2 (1 nmol/mL), CYP2A6 (2 nmol/mL), CYP2B6 (1 nmol/mL), CYP2C8 (1 nmol/mL), CYP2C9 (2 nmol/mL), CYP2C19 (1 nmol/mL), CYP2D6 (1 nmol/mL), CYP2E1 (2 nmol/mL), CYP3A4 (1 nmol/mL), CYP3A5 (1 nmol/mL), or flavin-containing monooxygenase 3 (FMO3, 5 mg/mL), as well as pooled human liver microsomes (pHLM, 20 mg protein/mL, 360 pmol total CYP/mg, 26 donors), pHLS9 (20 mg protein/mL, 8 donors), recombinant human carboxylesterase hCES1b (5 mg/mL), hCES1c (5 mg/mL), hCES2 (5 mg/mL), UGT reaction mixture solution A (25 mM UDP-glucuronic acid), and UGT reaction mixture solution B (250 mM Tris HCl, 40 mM MgCl_2_, and 125 μg alamethicin/mL) were obtained from Corning (Amsterdam, The Netherlands). After delivery, the enzymes were thawed at 37 °C, aliquoted, snap-frozen in liquid nitrogen, and stored at −80 °C until use. Acetonitrile (LC-MS grade), methanol (LC-MS grade), ammonium formate (analytical grade), formic acid (LC-MS grade), and all other reagents and chemicals (analytical grade) were bought from VWR (Darmstadt, Germany).

### 3.2. pHLS9 Incubations for Investigation of Phase I and II Metabolites

Incubations with pHLS9 were conducted in accordance with an earlier publication with minor modifications [9]. First, a solution with pHLS9 (2 mg protein/mL), 0.1 mM acetyl coenzyme A, 25 µg/mL alamethicin (UGT reaction mixture solution B), 90 mM phosphate buffer (pH 7.4), 2.5 mM Mg^2+^, 2.5 mM isocitrate, 0.6 mM NADP^+^, 0.9 U/mL isocitrate dehydrogenase, and 100 U/mL superoxide dismutase was preincubated for 10 min at 37 °C. After preincubation, 2.5 mM UDP-glucuronic acid (UGT reaction mixture solution A), 40 µM PAPS, 1.2 mM SAM, 1 mM dithiothreitol, 10 mM glutathione, and 25 µM QMPSB or QMPCB were added. All given concentrations are concentrations in the final incubation mixture (final incubation volume: 150 µL). The total amount of organic solvent in the incubations was less than 2% [16].

Reactions were started by adding the substrate (QMPSB or QMPCB). The duration of the incubation was set to six hours at 37 °C, with a 60 µL aliquot being taken after one hour.

The reactions of the aliquot were terminated by adding 20 µL ice-cold acetonitrile containing 2.5 µM trimipramine-d_3_ as internal standard (IS). After 360 min, metabolic reactions in the remaining incubation solution were terminated by the addition of 30 µL ice-cold acetonitrile containing 2.5 µM trimipramine-d_3_. All samples were cooled for 30 min at −20 °C and centrifuged at 18,407× *g* for 2 min. The supernatants were transferred to autosampler vials and analyzed using LC-HRMS/MS. Blank incubations (without substrate) and negative control incubations (without pHLS9) were carried out to confirm the absence of interfering and non-metabolically formed compounds. All incubations were performed in duplicates (*n* = 2).

### 3.3. Monooxygenase Activity Screening

Incubations with monooxygenases were performed in accordance with a previous study with minor modifications [17]. QMPSB or QMPCB (25 µM each) was incubated with CYP1A2, CYP2A6, CYP2B6, CYP2C8, CYP2C9, CYP2C19, CYP2D6, CYP2E1, CYP3A4, CYP3A5 (50 pmol/mL each), or FMO3 (0.25 mg protein/mL), as well as 90 mM phosphate buffer (pH 7.4), 5 mM Mg^2+^, 5 mM isocitrate, 1.2 mM NADP^+^, 0.5 U/mL isocitrate dehydrogenase, and 200 U/mL superoxide dismutase, for 30 min at 37 °C. For incubations with CYP2A6 and CYP2C9, 90 mM Tris buffer (pH 7.4) was used instead of phosphate buffer, according to the recommendations of the manufacturer. All given concentrations are concentrations in the final incubation mixture (final incubation volume: 50 µL). Reactions were started by adding the substrate (QMPSB or QMPCB). After 30 min, reactions were terminated by adding 50 µL of ice-cold acetonitrile containing 2.5 µM trimipramine-d_3_. The samples were centrifuged at 18,407× *g* for 2 min, and the supernatants were transferred to autosampler vials, and analyzed using LC-HRMS/MS. A negative control sample (without enzyme) was incubated to identify non-metabolically formed compounds. Furthermore, pHLM (1 mg/mL) positive control samples were incubated. All incubations were performed in duplicates (*n* = 2).

### 3.4. Esterase Activity Screening with Recombinant hCES1b, hCES1c, hCES2, pHLM, and pHLS9

Incubations with esterases were carried out as described in a previous publication with minor modifications [18]. QMPSB and QMPCB (10 µM final concentration) were incubated with hCES1b, hCES1c, hCES2 (0.2 µg/µL final concentration each), pHLM, and pHLS9 (2 µg/µL final concentration each) in phosphate buffer 100 mM pH 7.4 for 60 min at 37 °C. The total volume of the incubations was 150 μL. The incubations were started by adding the substrates. Negative control incubations (without enzyme) were conducted to evaluate non-enzymatic ester hydrolysis. At 0 and 60 min, 40 µL samples were taken. These samples were stopped immediately with 120 µL ice-cold acetonitrile containing trimipramine-d_3_ (2.5 µM). After centrifugation for 2 min at 18,407× *g*, 50 µL of the supernatant was transferred to an autosampler vial, and analyzed using LC-HRMS/MS. All incubations were performed in duplicates (*n* = 2).

### 3.5. LC-HRMS/MS Settings

A Thermo Fisher Scientific (TF, Dreieich, Germany) Dionex UltiMate 3000 RS LC system consisting of a degasser, a quaternary pump, and an HTC PAL autosampler (CTC Analytics AG, Zwingen, Switzerland) coupled to a TF Q-Exactive mass spectrometer with heated electrospray ionization (HESI)-II source was used. An external mass calibration was done prior to analysis according to the manufacturer’s recommendations. An injection volume of 5 µL was used for all samples. Gradient elution was implemented on a TF Accucore Phenyl-Hexyl column (100 mm × 2.1 mm, 2.6 µm) at 40 °C in accordance with previous work [19]. The mobile phases for gradient elution consisted of 2 mM aqueous ammonium formate containing formic acid (0.1%, *v*/*v*, pH 3, eluent A) and 2 mM ammonium formate solution with acetonitrile:methanol (1:1, *v*/*v*), water (1%, *v*/*v*), and formic acid (0.1%, *v*/*v*, eluent B). The following gradient settings were used: 0–1 min hold 99% A, 1–10 min to 1% A, 10–11.5 min hold 1% A, and 11.5–13.5 min hold 99% A. The flow rate was 500 μL/min (0–10 min) and 800 µL/min (10–13.5 min). The following HESI-II source settings were used: heater temperature, 320 °C; ion transfer capillary temperature, 320 °C; spray voltage, 4.0 kV; ionization mode, positive or negative; sheath gas, 60 arbitrary units (AU); auxiliary gas, 10 AU; sweep gas, 0 AU; S-lens RF level, 60.0. Mass spectrometry was carried out in full scan mode with subsequent data-dependent acquisition of MS^2^ (ddMS^2^) with priority to mass to charge ratios (*m/z*) of the parent compound and expected metabolites. The following full scan data acquisition settings were used: resolution, 35,000 FWHM at *m/z* 200; microscans, 1; automatic gain control (AGC) target, 1E6; maximum injection time (maxIT), 120 ms; scan range, *m/z* 80–850. An inclusion list containing *m/z* values of QMPSB or QMPCB and of expected metabolites such as hydrolysis products, dealkyl and hydroxy metabolites (phase I), as well as sulfates, and glucuronides (phase II), was used for ddMS^2^ mode. The following ddMS^2^ mode settings were used: option “pick others”, enabled; dynamic exclusion, feature not used; resolution, 17,500 FWHM at *m/z* 200; microscans, 1; isolation window, *m/z* 1.0; loop count, 5; AGC target, 2E5; maxIT, 250 ms; high collision dissociation cell with stepped normalized collision energy, 17.5, 35.0, 52.5; exclude isotopes, on; spectrum data type, profile. ChemSketch 2020.1.1 (ACD/Labs, Toronto, ON, Canada) was used to draw chemical structures of QMPSB, QMPCB, and their expected metabolites, and to calculate the exact masses. TF Xcalibur Qual Browser 4.1.31.9 was used for MS data analysis.

## 4. Conclusions

In total, 21 metabolites of QMPSB and 34 metabolites of QMPCB were tentatively identified using in vitro tools. All three tested human carboxylesterase isoforms (hCES1b, hCES1c, and hCES2) catalyzed the hydrolysis. The esterase activity screening also revealed a rapid non-enzymatic ester hydrolysis of both compounds. It is thus questionable, whether metabolites with an intact ester bond might be detectable in toxicological screenings. The ester hydrolysis products and their glucuronides and monohydroxy metabolites are thus recommended as targets for toxicological screenings for both substances. In the case of QMPSB, some of these targets could only be detected in negative ionization mode. Therefore, negative ionization mode is recommended for appropriate screenings. Different CYP isoforms were involved in the phase I metabolism of both substances, mainly CYP2B6, CYP2C8, CYP2C9, CYP2C19, CYP3A4, and CYP3A5. Due to the involvement of several CYP isozymes, the inhibition of a single CYP isozyme by a drug-drug interaction is unlikely to have a significant effect on hepatic clearance.

## Data Availability

The data presented in this study are available in this paper and the corresponding Electronic Supplementary Material.

## Supplementary Materials

The following are available online at https://www.mdpi.com/article/10.3390/metabo11080509/s1, Table S1: List of QMPSB and all detected QMPSB metabolites with incubation type and the ESI mode they were detected in, precursor ion (PI) and characteristic fragment ions (FI) masses in MS^2^, relative intensities in MS^2^, calculated exact masses, elemental composition, and deviation from measured to calculated masses, and retention time (RT), Table S2: List of QMPCB and all detected QMPCB metabolites with incubation type and the ESI mode they were detected in, precursor ion (PI) and characteristic fragment ions (FI) masses in MS^2^, relative intensities in MS^2^, calculated exact masses, elemental composition, and deviation from measured to calculated masses, and retention time (RT), Table S3: Detection of QMPSB metabolites in pHLS9 and monooxygenases activity screening incubations, Table S4: Detection of QMPCB metabolites in pHLS9 and monooxygenases activity screening incubations, Figure S1: Chromatograms of QMPSB and its five most abundant metabolites in pHLS9 incubation (6 h sample) in positive or negative ionization mode, Figure S2: Chromatograms of QMPCB and its five most abundant metabolites in pHLS9 incubation (6 h sample) in positive ionization mode, Figure S3: High-resolution MS^2^ spectra of QMPSB metabolites detected in pHLS9 and monooxygenases activity screening, Figure S4: High-resolution MS^2^ spectra of QMPCB metabolites detected in pHLS9 and monooxygenases activity screening.
