# Supplementary material for: In Vitro Metabolic Fate of the Synthetic Cannabinoid Receptor Agonists QMPSB and QMPCB (SGT-11) Including Isozyme Mapping and Esterase Activity
Source: Metabolites. 2021 Aug 3;11(8):509. doi: 10.3390/metabo11080509 (PMC8400906; doi:10.3390/metabo11080509)
Supplement: Supplementary file 1 [file metabolites-11-00509-s001.zip › metabolites-1293830-supplementary.pdf]

Supplementary materials

In Vitro Metabolic Fate of the Synthetic Cannabinoid Receptor Agonists QMPSB and QMPCB (SGT-11) Including Isozyme Mapping and Esterase Activity

Matthias J. Richter [1], Lea Wagmann [1], Tanja M. Gampfer [1], Simon D. Brandt [2] and Markus R. Meyer [1,*]

[1] Department of Experimental and Clinical Toxicology, Institute of Experimental and Clinical Pharmacologyand Toxicology, Center for Molecular Signaling (PZMS), Saarland University, 66421 Homburg, Germany; Matthias.Richter@uks.eu (M.J.R.), Lea.Wagmann@uks.eu (L.W.), Tanja.Gampfer@uks.eu (T.M.G.)

[2] School of Pharmacy and Biomolecular Sciences, Liverpool John Moores University, Liverpool L3 3AF, UK; s.brandt@ljmu.ac.uk

* Correspondence: markus.meyer@uks.eu; Tel.: +49-6841-16-26438

Table S1. List of QMPSB and all detected QMPSB metabolites with incubation type and the ESI mode they were detected in, precursor ion (PI) and characteristic fragment ions (FI) masses in MS^2, relative intensities in MS^2, calculated exact masses, elemental composition, and deviation from measured to calculated masses, and retention time (RT). pos, positive ionization mode; neg, negative ionization mode; gluc, glucuronic acid.

Metabolite ID	Metabolite	Incubation	ESI Mode	Measured Masses of Characteristic Ions, *m/z*		Relative Intensity in MS^2, %	Calculated Exact Masses, *m/z*	Elemental Composition	Error, ppm	RT, min
QMPSB	-	pHLS9/ CYP	pos	PI at *m/z*	411.1375	28	411.1373	C22H23O4N2S	0.48	9.4
				FI at *m/z*	266.0846	100	266.0845	C13H16O3NS	0.23	
				FI at *m/z*	183.0111	32	183.0110	C8H7O3S	0.33	
				FI at *m/z*	135.0442	26	135.0441	C8H7O2	1.07	
				FI at *m/z*	119.0494	41	119.0491	C8H7O	2.18	
				FI at *m/z*	91.0548	17	91.0542	C7H7	6.29	
SM1	*N,N*-bisdealkyl	CYP	pos	PI at *m/z*	343.0745	16	343.0747	C17H15O4N2S	-0.59	7.6
				FI at *m/z*	198.0220	100	198.0219	C8H8O3NS	0.30	
				FI at *m/z*	180.9954	29	180.9954	C8H5O3S	0.06	
				FI at *m/z*	153.0005	16	153.0005	C7H5O2S	0.16	
SM2	dehydro (isomer 1)	CYP	pos	PI at *m/z*	409.1218	22	409.1217	C22H21O4N2S	0.36	8.8
				FI at *m/z*	264.0689	100	264.0689	C13H14O3NS	0.04	
				FI at *m/z*	183.0111	14	183.0110	C8H7O3S	0.33	
				FI at *m/z*	83.0736	45	83.0730	C5H9N	7.81	
SM3	dehydro (isomer 2)	CYP	pos	PI at *m/z*	409.1219	25	409.1217	C22H21O4N2S	0.60	9.5
				FI at *m/z*	264.0690	100	264.0689	C13H14O3NS	0.42	
				FI at *m/z*	183.0112	14	183.0110	C8H7O3S	0.87	
				FI at *m/z*	83.0736	43	83.0730	C5H9N	7.81	
SM4	hydroxy + dehydro	CYP	pos	PI at *m/z*	425.1169	31	425.1166	C22H21O5N2S	0.78	8.1
				FI at *m/z*	264.0693	100	264.0689	C13H14O3NS	1.55	
				FI at *m/z*	183.0112	15	183.0110	C8H7O3S	0.87	
				FI at *m/z*	119.0496	12	119.0491	C8H7O	3.86	
				FI at *m/z*	83.0737	47	83.0730	C5H9ON	9.02	
SM5	hydroxy (isomer 1)	CYP	pos	PI at *m/z*	427.1328	62	427.1322	C22H23O5N2S	1.36	8.2
				FI at *m/z*	300.0901	100	300.0900	C13H16O5NS	0.27	
				FI at *m/z*	282.0796	38	282.0795	C13H16O4NS	0.52	
				FI at *m/z*	199.0061	39	199.0060	C8H7O4S	0.73	
				FI at *m/z*	163.0503	77	163.0502	C8H7O2N2	0.59	
				FI at *m/z*	135.0442	83	135.0441	C8H7O2	1.07	
				FI at *m/z*	107.0496	43	107.0491	C7H7O	4.29	
				FI at *m/z*	91.0548	26	91.0542	C7H7	6.29	

Table S1. (continued)

Metabolite ID	Metabolite	Incubation	ESI Mode	Measured Masses of Characteristic Ions, *m/z*		Relative Intensity in MS², %	Calculated Exact Masses, *m/z*	Elemental Composition	Error, ppm	RT, min
SM6	hydroxy (isomer 2)	CYP	pos	PI at *m/z*	427.1326	41	427.1322	C22H23O5N2S	0.89	8.8
				FI at *m/z*	266.0850	100	266.0845	C13H16O3NS	1.73	
				FI at *m/z*	183.0113	31	183.0110	C8H7O3S	1.42	
				FI at *m/z*	135.0443	23	135.0441	C8H7O2	1.81	
				FI at *m/z*	119.0496	38	119.0491	C8H7O	3.86	
				FI at *m/z*	91.0549	15	91.0542	C7H7	7.39	
SM7	dihydoxy (isomer 1)	CYP	pos	PI at *m/z*	443.1276	50	443.1271	C22H23O6N2S	1.05	8.1
				FI at *m/z*	298.0745	37	298.0744	C13H16O5NS	0.44	
				FI at *m/z*	199.0060	100	199.0060	C8H7O4S	0.23	
				FI at *m/z*	163.0503	54	163.0502	C8H7O2N2	0.59	
				FI at *m/z*	135.0442	73	135.0441	C8H7O2	1.07	
				FI at *m/z*	107.0496	57	107.0491	C7H7O	4.29	
				FI at *m/z*	82.0658	31	82.0651	C5H8N	8.21	
SM8	dihydoxy (isomer 2)	CYP	pos	PI at *m/z*	443.1290	28	443.1271	C22H23O6N2S	4.21	8.1
				FI at *m/z*	264.0695	100	264.0689	C13H14O3NS	2.31	
				FI at *m/z*	183.0113	16	183.0110	C8H7O3S	1.42	
				FI at *m/z*	162.0554	50	162.0550	C9H8O2N	2.75	
				FI at *m/z*	83.0738	47	83.0730	C5H9N	10.22	
SM9	ester hydrolysis (carboxylic acid)	pHLS9/ CYP	pos	PI at *m/z*	284.0952	47	284.0951	C13H18O4NS	0.34	7.9
				FI at *m/z*	135.0442	8	135.0441	C8H7O2	1.07	
				FI at *m/z*	85.0892	37	85.0886	C5H11N	7.04	
				FI at *m/z*	84.0814	100	84.0808	C5H10N	7.42	
		pHLS9/ CYP	neg	PI at *m/z*	282.0805	20	282.0806	C13H16O4NS	-0.18	
				FI at *m/z*	238.0904	8	238.0907	C12H16O2NS	-1.35	
				FI at *m/z*	148.0426	100	148.0438	C5H10O2NS	-7.92	
				FI at *m/z*	63.9610	13	63.9624	O2S	-22.64	
SM10	ester hydrolysis (carboxylic acid) + *N,N*-bisdealkyl	CYP	neg	PI at *m/z*	214.0173	23	214.0180	C8H8O4NS	-3.04	5.0
				FI at *m/z*	170.0271	48	170.0281	C7H8O2NS	-6.01	
				FI at *m/z*	79.9797	100	79.981172	H2O2NS	-18.40	
SM11	ester hydrolysis (carboxylic acid) + dehydro	pHLS9/ CYP	neg	PI at *m/z*	280.0642	34	280.0649	C13H14O4NS	-2.51	8.0
				FI at *m/z*	146.0269	100	146.0281	C5H8O2NS	-8.37	
				FI at *m/z*	63.9610	39	63.9624	O2S	-22.64	
SM12	ester hydrolysis (carboxylic acid) + hydroxy (isomer 1)	pHLS9/ CYP	neg	PI at *m/z*	298.0751	47	298.0755	C13H16O5NS	-1.23	6.4
				FI at *m/z*	155.0161	100	155.0172	C7H7O2S	-7.24	
				FI at *m/z*	63.9611	14	63.9624	O2S	-21.07	

Table S1. (continued)

Meta-bolite ID	Metabolite	Incubation	ESI Mode	Measured Masses of Characteristic Ions, *m/z*		Relative Intensity in MS², %	Calculated Exact Masses, *m/z*	Elemental Composition	Error, ppm	RT, min
SM13	ester hydrolysis (carboxylic acid) + hydroxy (isomer 2)	CYP	neg	PI at *m/z*	298.0758	44	298.0755	C13H16O5NS	1.12	6.6
				FI at *m/z*	148.0425	100	148.0438	C5H10O2NS	-8.59	
SM14	ester hydrolysis (carboxylic acid) + hydroxy (isomer 3)	CYP	neg	PI at *m/z*	298.0753	82	298.0755	C13H16O5NS	-0.56	7.0
				FI at *m/z*	254.0855	100	254.0856	C12H16O3NS	-0.54	
				FI at *m/z*	170.0271	94	170.0281	C7H8O2NS	-6.01	
				FI at *m/z*	155.0159	35	155.0172	C7H7O2S	-8.53	
				FI at *m/z*	100.0753	33	100.0768	C5H10ON	-14.87	
SM15	ester hydrolysis (carboxylic acid) + dihydroxy	pHLS9/ CYP	neg	PI at *m/z*	314.0702	41	314.0704	C13H16O6NS	-0.58	6.2
				FI at *m/z*	270.0801	4	270.0806	C12H16O4NS	-1.67	
				FI at *m/z*	170.0270	100	170.0281	C7H8O2NS	-6.60	
SM16	ester hydrolysis (carboxylic acid) + gluc	pHLS9	pos	PI at *m/z*	Not detected	0	460.1272	C19H26O10NS	-	6.9
				FI at *m/z*	284.0949	100	284.0951	C13H18O4NS	-0.72	
				FI at *m/z*	135.0443	7	135.0441	C8H7O2	1.81	
				FI at *m/z*	85.0892	32	85.0886	C5H11N	7.04	
				FI at *m/z*	84.0814	90	84.0808	C5H10N	7.42	
		pHLS9	neg	PI at *m/z*	458.1134	3	458.1126	C19H24O10NS	1.66	
				FI at *m/z*	282.0807	47	282.0806	C13H16O4NS	0.52	
				FI at *m/z*	238.0909	14	238.0907	C12H16O2NS	0.75	
				FI at *m/z*	148.0425	100	148.0438	C5H10O2NS	-8.59	
				FI at *m/z*	113.0231	41	113.0244	C5H5O3	-11.66	
				FI at *m/z*	85.0281	38	85.0295	C4H5O2	-16.50	
SM17	ester hydrolysis (carboxylic acid) + dihydroxy + gluc	pHLS9	neg	PI at *m/z*	Not detected	0	490.1025	C19H24O12NS	-	5.6
				FI at *m/z*	314.0704	73	314.0704	C13H16O6NS	0.06	
				FI at *m/z*	170.0271	100	170.0281	C7H8O2NS	-6.01	
SM18	ester hydrolysis (8-hydroxyquinoline)	pHLS9/ CYP	pos	PI at *m/z*	146.0603	100	146.0600	C9H8ON	1.78	2.2
				FI at *m/z*	118.0655	11	118.0651	C8H8N	3.17	
SM19	ester hydrolysis (8-hydroxyquinoline) + hydroxy	CYP	pos	PI at *m/z*	162.0551	100	162.0550	C9H8O2N	0.89	1.3
				FI at *m/z*	134.0602	7	134.0600	C8H8ON	1.19	
SM20	ester hydrolysis (8-hydroxyquinoline) + sulfate	pHLS9	pos	PI at *m/z*	226.0169	12	226.0169	C9H8O4NS	0.20	2.2
				FI at *m/z*	146.0601	100	146.0600	C9H8ON	0.41	
				FI at *m/z*	133.0286	26	133.0284	C8H5O2	1.46	
SM21	ester hydrolysis (8-hydroxyquinoline) + gluc	pHLS9	pos	PI at *m/z*	322.0919	11	322.0921	C15H16O7N	-0.71	1.5
				FI at *m/z*	146.0601	100	146.0600	C9H8ON	0.41	
				FI at *m/z*	113.0236	4	113.0233	C5H5O3	2.48	
				FI at *m/z*	85.0290	7	85.0295	C4H5O2	-5.92	

Table S2. List of QMPCB and all detected QMPCB metabolites with incubation type and the ESI mode they were detected in, precursor ion (PI) and characteristic fragment ions (FI) masses in MS^2, relative intensities in MS^2, calculated exact masses, elemental composition, and deviation from measured to calculated masses, and retention time (RT). pos, positive ionization mode; neg, negative ionization mode; gluc, glucuronic acid.

Meta-bolite ID	Metabolite	Incuba tion	ESI Mode	Measured Masses of Characteristic Ions, *m/z*		Relative Intensity in MS^2, %	Calculated Exact Masses, *m/z*	Elemental Composition	Error, ppm	RT, min
QMPCB	-	pHLS9/ CYP	pos	PI at *m/z*	375.1696	8	375.1703	C23H23O3N2	-1.92	8.7
				FI at *m/z*	230.1172	100	230.1176	C14H16O2N	-1.54	
				FI at *m/z*	147.0437	13	147.0441	C9H7O2	-2.42	
				FI at *m/z*	145.0282	73	145.0284	C9H5O2	-1.42	
				FI at *m/z*	117.0336	5	117.0335	C8H5O	0.93	
				FI at *m/z*	89.0390	3	89.0386	C7H5	4.75	
CM1	*N,N*-bisdealkyl	CYP	pos	PI at *m/z*	307.1073	6	307.1077	C18H15O3N2	-1.36	7.2
				FI at *m/z*	162.0547	100	162.0550	C9H8O2N	-1.57	
				FI at *m/z*	145.0282	50	145.0284	C9H5O2	-1.42	
				FI at *m/z*	117.0335	4	117.0335	C8H5O	0.08	
CM2	carboxamide cleavage (carboxylic acid)	CYP	pos	PI at *m/z*	308.0916	6	308.0917	C18H14O4N	-0.43	8.0
				FI at *m/z*	163.0387	100	163.0390	C9H7O3	-1.66	
				FI at *m/z*	135.0439	11	135.0441	C8H7O2	-1.16	
CM3	*N,N*-bisdealkyl + hydroxy	CYP	pos	PI at *m/z*	323.1021	16	323.1026	C18H15O4N2	-1.65	6.5
				FI at *m/z*	178.0497	100	178.0499	C9H8O3N	-0.95	
				FI at *m/z*	161.0231	69	161.0233	C9H5O3	-1.37	
				FI at *m/z*	133.0286	16	133.0284	C8H5O2	1.46	
CM4	hydroxy + didehydro	CYP	pos	PI at *m/z*	387.1337	21	387.1339	C23H19O4N2	-0.60	8.1
				FI at *m/z*	242.0811	100	242.0812	C14H12O3N	-0.29	
				FI at *m/z*	145.0284	83	145.0284	C9H5O2	-0.04	
				FI at *m/z*	117.0339	9	117.0335	C8H5O	3.49	
CM5	hydroxy + dehydro (isomer 1)	CYP	pos	PI at *m/z*	389.1493	5	389.1496	C23H21O4N2	-0.73	7.1
				FI at *m/z*	371.1382	18	371.1390	C23H19O3N2	-2.21	
				FI at *m/z*	308.0909	26	308.0917	C18H14O4N	-2.71	
				FI at *m/z*	244.0965	62	244.0968	C14H14O3N	-1.31	
				FI at *m/z*	226.0860	21	226.0863	C14H12O2N	-1.13	
				FI at *m/z*	163.0387	61	163.0390	C9H7O3	-1.66	
				FI at *m/z*	145.0282	100	145.0284	C9H5O2	-1.42	
CM6	hydroxy + dehydro (isomer 2)	CYP	pos	PI at *m/z*	389.1497	16	389.1496	C23H21O4N2	0.30	7.4
				FI at *m/z*	228.1016	99	228.1019	C14H14O2N	-1.34	
				FI at *m/z*	145.0283	100	145.0284	C9H5O2	-0.73	
				FI at *m/z*	117.0336	8	117.0335	C8H5O	0.93	

Table S2. (continued)

Meta-bolite ID	Metabolite	Incubation	ESI Mode	Measured Masses of Characteristic Ions, *m/z*		Relative Intensity in MS², %	Calculated Exact Masses, *m/z*	Elemental Composition	Error, ppm	RT, min
CM7	hydroxy + dehydro (isomer 3)	CYP	pos	PI at *m/z*	389.1492	22	389.1496	C23H21O4N2	-0.99	8.6
				FI at *m/z*	244.0962	100	244.0968	C14H14O3N	-2.54	
				FI at *m/z*	161.0231	19	161.0233	C9H5O3	-1.37	
				FI at *m/z*	105.0336	26	105.0335	C7H5O	1.04	
				FI at *m/z*	84.0811	18	84.0808	C5H10N	3.85	
CM8	hydroxy (isomer 1)	CYP	pos	PI at *m/z*	391.1645	10	391.1652	C23H23O4N2	-1.88	7.3
				FI at *m/z*	246.1121	100	246.1125	C14H16O3N	-1.50	
				FI at *m/z*	145.0282	76	145.0284	C9H5O2	-1.42	
				FI at *m/z*	117.0336	6	117.0335	C8H5O	0.93	
CM9	hydroxy (isomer 2)	CYP	pos	PI at *m/z*	391.1671	4	391.1652	C25H29O3N	4.77	7.3
				FI at *m/z*	246.1123	38	246.1125	C14H16O3N	-0.69	
				FI at *m/z*	149.0233	100	149.0233	C8H5O3	-0.13	
				FI at *m/z*	71.0863	12	71.0855	C5H11	10.87	
CM10	hydroxy (isomer 3)	CYP	pos	PI at *m/z*	391.1648	14	391.1652	C23H23O4N2	-1.11	7.6
				FI at *m/z*	264.1227	100	264.1230	C14H18O4N	-1.26	
				FI at *m/z*	246.1122	30	246.1125	C14H16O3N	-1.10	
				FI at *m/z*	163.0386	16	163.0390	C9H7O3	-2.27	
				FI at *m/z*	145.0282	90	145.0284	C9H5O2	-1.42	
				FI at *m/z*	117.0337	7	117.0335	C8H5O	1.79	
CM11	hydroxy (isomer 4)	CYP	pos	PI at *m/z*	391.1645	12	391.1652	C23H23O4N2	-1.88	8.0
				FI at *m/z*	230.1172	100	230.1176	C14H16O2N	-1.54	
				FI at *m/z*	145.0282	76	145.0284	C9H5O2	-1.42	
				FI at *m/z*	117.0336	5	117.0335	C8H5O	0.93	
CM12	dihydroxy + dehydro (isomer 1)	CYP	pos	PI at *m/z*	405.1437	19	405.1445	C23H21O5N2	-1.97	7.2
				FI at *m/z*	308.0910	13	308.0917	C18H14O4N	-2.38	
				FI at *m/z*	163.0386	100	163.0390	C9H7O3	-2.27	
				FI at *m/z*	145.0281	52	145.0284	C9H5O2	-2.11	
				FI at *m/z*	117.0335	6	117.0335	C8H5O	0.08	
CM13	dihydroxy + dehydro (isomer 2)	CYP	pos	PI at *m/z*	405.1437	24	405.1445	C23H21O5N2	-1.97	7.6
				FI at *m/z*	278.1022	100	278.1023	C14H16O5N	-0.36	
				FI at *m/z*	260.0922	3	260.0917	C14H14O4N	1.79	
				FI at *m/z*	163.0387	10	163.0390	C9H7O3	-1.66	
				FI at *m/z*	145.0284	67	145.0284	C9H5O2	-0.04	
				FI at *m/z*	117.0334	6	117.0335	C8H5O	-0.78	

Table S2. (continued)

Meta-bolite ID	Metabolite	Incubation	ESI Mode	Measured Masses of Characteristic Ions, *m/z*		Relative Intensity in MS², %	Calculated Exact Masses, *m/z*	Elemental Composition	Error, ppm	RT, min
CM14	dihydroxy (isomer 1)	CYP	pos	PI at *m/z*	407.1592	15	407.1601	C23H23O5N2	-2.33	6.6
				FI at *m/z*	246.1122	100	246.1125	C14H16O3N	-1.10	
				FI at *m/z*	145.0282	78	145.0284	C9H5O2	-1.42	
				FI at *m/z*	117.0336	6	117.0335	C8H5O	0.93	
CM15	dihydroxy (isomer 2)	CYP	pos	PI at *m/z*	407.1594	19	407.1601	C23H23O5N2	-1.84	6.9
				FI at *m/z*	264.1229	100	264.1230	C14H18O4N	-0.51	
				FI at *m/z*	246.1119	16	246.1125	C14H16O3N	-2.32	
				FI at *m/z*	163.0390	15	163.0390	C9H7O3	0.18	
				FI at *m/z*	145.0283	94	145.0284	C9H5O2	-0.73	
				FI at *m/z*	117.0337	8	117.0335	C8H5O	1.79	
CM16	dihydroxy (isomer 3)	CYP	pos	PI at *m/z*	407.1596	9	407.1601	C23H23O5N2	-1.35	7.6
				FI at *m/z*	262.1062	1	262.1074	C14H16O4N	-4.52	
				FI at *m/z*	163.0387	100	163.0390	C9H7O3	-1.66	
				FI at *m/z*	145.0282	9	145.0284	C9H5O2	-1.42	
CM17	trihydroxy	CYP	pos	PI at *m/z*	423.1554	14	423.1551	C23H23O6N2	0.80	7.1
				FI at *m/z*	278.1025	14	278.1023	C14H16O5N	0.72	
				FI at *m/z*	163.0388	100	163.0390	C9H7O3	-1.04	
				FI at *m/z*	100.0762	19	100.0757	C5H10ON	5.10	
CM18	ester hydrolysis (carboxylic acid)	pHLS9/ CYP	pos	PI at *m/z*	248.1281	100	248.1281	C14H18O3N	-0.08	7.0
				FI at *m/z*	163.0389	67	163.0390	C9H7O3	-0.43	
				FI at *m/z*	112.0760	7	112.0757	C6H10ON	2.77	
				FI at *m/z*	69.0706	10	69.0699	C5H9	10.47	
		pHLS9/ CYP	neg	PI at *m/z*	246.1131	71	246.1136	C14H16O3N	-1.90	
				FI at *m/z*	202.1228	100	202.1237	C13H16ON	-4.64	
CM19	ester hydrolysis (carboxylic acid) + *N,N*-bisdealkyl	CYP	pos	PI at *m/z*	180.0654	100	180.0655	C9H10O3N	-0.67	0.6
				FI at *m/z*	162.0547	69	162.0550	C9H8O2N	-1.57	
				FI at *m/z*	134.0599	24	134.0600	C8H8ON	-1.04	
CM20	ester hydrolysis (carboxylic acid) + dehydro (isomer 1)	CYP	pos	PI at *m/z*	246.1118	3	246.1125	C14H16O3N	-2.72	6.3
				FI at *m/z*	163.0387	100	163.0390	C8H7O3	-1.66	
				FI at *m/z*	135.0440	15	135.0441	C8H7O2	-0.41	
CM21	ester hydrolysis (carboxylic acid) + dehydro (isomer 2)	CYP	pos	PI at *m/z*	246.1121	41	246.1125	C14H16O3N	-1.50	7.3
				FI at *m/z*	145.0282	100	145.0284	C9H5O2	-1.42	
				FI at *m/z*	117.0336	20	117.0335	C8H5O	0.93	
		CYP	neg	PI at *m/z*	244.0971	55	244.0979	C14H14O3N	-3.35	
				FI at *m/z*	200.1070	100	200.1081	C13H14ON	-5.44	

Table S2. (continued)

Meta-bolite ID	Metabolite	Incubation	ESI Mode	Measured Masses of Characteristic Ions, *m/z*		Relative Intensity in MS^2, %	Calculated Exact Masses, *m/z*	Elemental Composition	Error, ppm	RT, min
CM22	ester hydrolysis (carboxylic acid) + hydroxy + dehydro	CYP	pos	PI at *m/z*	262.1070	18	262.1074	C14H16O4N	-1.47	5.4
				FI at *m/z*	163.0387	100	163.0390	C9H7O3	-1.66	
				FI at *m/z*	135.0439	10	135.0441	C8H7O2	-1.16	
				FI at *m/z*	107.0493	6	107.0491	C7H7O	1.49	
CM23	ester hydrolysis (carboxylic acid) + hydroxy (isomer 1)	pHLS9/ CYP	pos	PI at *m/z*	264.1231	29	264.1230	C14H18O4N	0.25	5.4
				FI at *m/z*	163.0390	100	163.0390	C9H7O3	0.18	
				FI at *m/z*	135.0442	7	135.0441	C8H7O2	1.07	
				FI at *m/z*	91.0549	11	91.0542	C7H7	7.39	
		pHLS9/ CYP	neg	PI at *m/z*	262.1076	57	262.1085	C14H16O4N	-3.37	
				FI at *m/z*	134.0596	100	134.0611	C8H8ON	-11.46	
				FI at *m/z*	98.0596	2	98.0611	C5H8ON	-15.67	
CM24	ester hydrolysis (carboxylic acid) + hydroxy (isomer 2)	CYP	pos	PI at *m/z*	264.1226	21	264.1230	C14H18O4N	-1.64	5.7
				FI at *m/z*	246.1123	54	246.1125	C14H16O3N	-0.69	
				FI at *m/z*	163.0388	100	163.0390	C9H7O3	-1.04	
				FI at *m/z*	135.0442	9	135.0441	C8H7O2	1.07	
				FI at *m/z*	84.0812	13	84.0808	C5H10N	5.04	
		CYP	neg	PI at *m/z*	262.1080	100	262.1085	C14H16O4N	-1.84	
				FI at *m/z*	218.1172	21	218.1187	C13H16O2N	-6.66	
				FI at *m/z*	148.0753	39	148.0768	C9H10ON	-10.05	
				FI at *m/z*	119.0487	50	119.0502	C8H7O	-12.92	
				FI at *m/z*	91.0538	11	91.0553	C7H7	-16.74	
CM25	ester hydrolysis (carboxylic acid) + hydroxy (isomer 3)	pHLS9/ CYP	pos	PI at *m/z*	264.1229	2	264.1230	C14H18O4N	-0.51	6.3
				FI at *m/z*	246.1122	10	246.1125	C14H16O3N	-1.10	
				FI at *m/z*	163.0387	100	163.0390	C9H7O3	-1.66	
				FI at *m/z*	135.0440	9	135.0441	C8H7O2	-0.41	
				FI at *m/z*	84.0813	5	84.0808	C5H10N	6.23	
		pHLS9/ CYP	neg	PI at *m/z*	262.1082	100	262.1085	C14H16O4N	-1.08	
				FI at *m/z*	218.1179	29	218.1187	C13H16O2N	-3.45	
				FI at *m/z*	134.0597	18	134.0611	C8H8ON	-10.72	
				FI at *m/z*	119.0488	96	119.0502	C8H7O	-12.08	
				FI at *m/z*	91.0538	11	91.0553	C7H7	-16.74	
CM26	ester hydrolysis (carboxylic acid) + dihydroxy (isomer 1)	CYP	pos	PI at *m/z*	Not detected	0	280.1179	C14H18O5N	-	5.0
				FI at *m/z*	262.1075	18	262.1074	C14H16O4N	0.44	
				FI at *m/z*	163.0385	100	163.0390	C9H7O3	-2.88	
				FI at *m/z*	149.0232	61	149.0233	C8H5O3	-0.81	

Table S2. (continued)

Metabolite ID	Metabolite	Incubation	ESI Mode	Measured Masses of Characteristic Ions, *m/z*		Relative Intensity in MS², %	Calculated Exact Masses, *m/z*	Elemental Composition	Error, ppm	RT, min
CM27	ester hydrolysis (carboxylic acid) + dihydroxy (isomer 2)	CYP	neg	PI at *m/z*	278.1021	39	278.1034	C14H16O5N	-4.66	5.0
				FI at *m/z*	178.0499	75	178.0510	C9H8O3N	-5.99	
				FI at *m/z*	134.0597	100	134.0611	C8H8ON	-10.72	
CM28	ester hydrolysis (carboxylic acid) + dihydroxy (isomer 3)	pHLS9/ CYP	neg	PI at *m/z*	278.1031	100	278.1034	C14H16O5N	-1.06	5.7
				FI at *m/z*	260.0924	26	260.0928	C14H14O4N	-1.66	
				FI at *m/z*	216.1023	44	216.1030	C13H14O2N	-3.25	
				FI at *m/z*	198.0912	24	198.0924	C13H12ON	-6.25	
				FI at *m/z*	134.0596	28	134.0611	C8H8ON	-11.46	
				FI at *m/z*	91.0538	19	91.0553	C7H7	-16.74	
CM29	ester hydrolysis (carboxylic acid) + gluc	pHLS9	pos	PI at *m/z*	424.1601	5	424.1602	C20H26O9N	-0.25	6.1
				FI at *m/z*	248.1281	100	248.1281	C14H18O3N	-0.08	
				FI at *m/z*	230.1176	31	230.1176	C14H16O2N	0.20	
				FI at *m/z*	163.0389	30	163.0390	C9H7O3	-0.43	
				FI at *m/z*	145.0284	15	145.0284	C9H5O2	-0.04	
				FI at *m/z*	112.0760	4	112.0757	C6H10ON	2.77	
				FI at *m/z*	69.0706	4	69.0699	C5H9	10.47	
		pHLS9	neg	PI at *m/z*	422.1459	6	422.1457	C20H24O9N	0.58	
				FI at *m/z*	246.1131	100	246.1136	C14H16O3N	-1.90	
				FI at *m/z*	202.1228	88	202.1237	C13H16ON	-4.64	
				FI at *m/z*	175.0236	12	175.0248	C6H7O6	-6.92	
				FI at *m/z*	113.0229	43	113.0244	C5H5O3	-13.43	
				FI at *m/z*	85.0280	41	85.0295	C4H5O2	-17.68	
CM30	ester hydrolysis (8-hydroxyquinoline)	pHLS9/ CYP	pos	PI at *m/z*	146.0603	100	146.0600	C9H8ON	1.78	2.2
				FI at *m/z*	118.0654	6	118.0651	C8H8N	2.32	
CM31	ester hydrolysis (8-hydroxyquinoline) + hydroxy	CYP	pos	PI at *m/z*	162.0547	100	162.0550	C9H8O2N	-1.57	1.2
				FI at *m/z*	134.0599	7	134.0600	C8H8ON	-1.04	
CM32	ester hydrolysis (8-hydroxyquinoline) + sulfate	pHLS9	pos	PI at *m/z*	226.0169	10	226.0169	C9H8O4NS	0.20	2.2
				FI at *m/z*	146.0601	100	146.0600	C9H8ON	0.41	
CM33	ester hydrolysis (8-hydroxyquinoline) + gluc	pHLS9	pos	PI at *m/z*	322.0919	10	322.0921	C15H16O7N	-0.71	1.5
				FI at *m/z*	146.0600	100	146.0600	C9H8ON	-0.27	
				FI at *m/z*	113.0237	4	113.0233	C5H5O3	3.36	
CM34	ester hydrolysis (8-hydroxyquinoline) + hydroxy + gluc	pHLS9	pos	PI at *m/z*	338.0864	12	338.0870	C15H16O8N	-1.90	0.9
				FI at *m/z*	162.0550	100	162.0550	C9H8O2N	0.28	

Table S3. Detection of QMPSB metabolites in pHLS9 and monooxygenases activity screening incubations (chemical structures are given in Figure 2). SM, metabolites of QMPSB; CYP, cytochrome P450; FMO, flavin-containing monooxygenase; pHLM, pooled human liver microsomes; +, metabolite detected; -, metabolite not detected; gluc, glucuronic acid.

**Metabolite**		**pHLS9**	**CYP**										**FMO3**	**pHLM**
			**1A2**	**2A6**	**2B6**	**2C8**	**2C9**	**2C19**	**2D6**	**2E1**	**3A4**	**3A5**		
SM1	*N,N*-bisdealkyl	-	-	-	-	+	-	+	-	-	+	+	-	-
SM2	dehydro isomer 1	-	-	-	-	+	+	+	-	-	+	+	-	+
SM3	dehydro isomer 2	-	-	-	-	+	+	+	-	-	+	+	-	+
SM4	hydroxy + dehydro	-	-	-	-	+	+	+	-	-	-	-	-	-
SM5	hydroxy isomer 1	-	-	-	+	+	+	+	-	-	-	-	-	-
SM6	hydroxy isomer 2	-	+	-	-	+	+	+	+	-	-	-	-	-
SM7	dihydoxy isomer 1	-	-	-	-	+	-	+	-	-	+	+	-	-
SM8	dihydoxy isomer 2	-	+	-	-	+	+	+	-	-	-	-	-	-
SM9	ester hydrolysis (carboxylic acid)	+	+	+	+	+	+	+	+	+	+	+	+	+
SM10	ester hydrolysis (carboxylic acid) + *N,N*-bisdealkyl	-	-	-	-	-	-	-	-	-	+	+	-	-
SM11	ester hydrolysis (carboxylic acid) + dehydro	+	-	-	-	+	+	+	-	-	+	+	-	+
SM12	ester hydrolysis (carboxylic acid) + hydroxy (isomer 1)	+	+	-	+	+	+	+	-	-	-	+	-	+
SM13	ester hydrolysis (carboxylic acid) + hydroxy (isomer 2)	-	+	-	-	-	-	-	-	-	+	-	-	-
SM14	ester hydrolysis (carboxylic acid) + hydroxy (isomer 3)	-	-	-	-	+	+	+	-	-	+	+	-	+
SM15	ester hydrolysis (carboxylic acid) + dihydroxy	+	-	-	-	-	-	-	-	-	+	-	-	+
SM16	ester hydrolysis (carboxylic acid) + gluc	+	-	-	-	-	-	-	-	-	-	-	-	-
SM17	ester hydrolysis (carboxylic acid) + dihydroxy + gluc	+	-	-	-	-	-	-	-	-	-	-	-	-
SM18	ester hydrolysis (8-hydroxyquinolin)	+	+	+	+	+	+	+	+	+	+	+	+	+
SM19	ester hydrolysis (8-hydroxyquinolin) + hydroxy	-	+	+	+	+	+	+	+	+	+	+	+	+
SM20	ester hydrolysis (8-hydroxyquinolin) + sulfate	+	-	-	-	-	-	-	-	-	-	-	-	-
SM21	ester hydrolysis (8-hydroxyquinolin) + gluc	+	-	-	-	-	-	-	-	-	-	-	-	-

Table S4. Detection of QMPCB metabolites in pHLS9 and monooxygenases activity screening incubations (chemical structures are given in Figure 2). CM, metabolites of QMPCB; CYP, cytochrome P450; FMO, flavin-containing monooxygenase; pHLM, pooled human liver microsomes; +, metabolite detected; -, metabolite not detected; gluc, glucuronic acid.

**Metabolite**		**pHLS9**	**CYP**										**FMO3**	**pHLM**
			**1A2**	**2A6**	**2B6**	**2C8**	**2C9**	**2C19**	**2D6**	**2E1**	**3A4**	**3A5**		
CM1	*N,N*-bisdealkyl	-	-	-	-	-	-	-	-	-	+	+	-	+
CM2	carboxamide cleavage (carboxylic acid)	-	-	-	-	-	-	-	-	-	+	+	-	-
CM3	*N,N*-bisdealkyl + hydroxy	-	-	-	-	-	-	-	-	-	+	-	-	-
CM4	hydroxy + didehydro	-	-	-	-	-	-	-	-	-	+	+	-	-
CM5	hydroxy + dehydro (isomer 1)	-	-	-	-	-	+	+	-	-	+	+	-	+
CM6	hydroxy + dehydro (isomer 2)	-	-	-	-	+	-	+	-	-	-	-	-	-
CM7	hydroxy + dehydro (isomer 3)	-	-	+	-	-	+	-	-	-	+	+	-	-
CM8	hydroxy (isomer 1)	-	-	-	+	+	+	+	-	-	-	-	-	-
CM9	hydroxy (isomer 2)	-	+	-	-	-	-	-	+	+	+	-	-	+
CM10	hydroxy (isomer 3)	-	-	-	+	+	+	+	-	-	-	+	-	-
CM11	hydroxy (isomer 4)	-	+	-	-	+	+	+	+	-	-	-	-	-
CM12	dihydroxy + dehydro (isomer 1)	-	-	-	-	-	-	-	-	-	+	+	-	+
CM13	dihydroxy + dehydro (isomer 2)	-	-	-	-	-	-	-	-	-	+	+	-	-
CM14	dihydroxy (isomer 1)	-	-	-	-	+	-	+	-	-	-	-	-	-
CM15	dihydroxy (isomer 2)	-	-	-	-	+	-	-	-	-	-	-	-	-
CM16	dihydroxy (isomer 3)	-	-	-	-	+	-	+	-	-	+	+	-	+
CM17	trihydroxy	-	-	-	-	-	-	-	-	-	+	+	-	+
CM18	ester hydrolysis (carboxylic acid)	+	+	+	+	+	+	+	+	+	+	+	+	+
CM19	ester hydrolysis (carboxylic acid) + *N,N*-bisdealkyl	-	+	-	-	+	-	-	-	-	-	-	-	+
CM20	ester hydrolysis (carboxylic acid) + dehydro (isomer 1)	-	-	-	-	-	-	-	-	-	+	+	-	+
CM21	ester hydrolysis (carboxylic acid) + dehydro (isomer 2)	-	-	-	+	+	+	+	-	-	+	+	-	+
CM22	ester hydrolysis (carboxylic acid) + hydroxy + dehydro	-	-	-	-	-	-	-	-	-	+	+	-	+
CM23	ester hydrolysis (carboxylic acid) + hydroxy (isomer 1)	+	-	-	+	+	+	+	-	-	+	+	-	+

Table S4. (continued)

Metabolite		pHLS9	CYP										FMO3	pHLM
			1A2	2A6	2B6	2C8	2C9	2C19	2D6	2E1	3A4	3A5		
CM24	ester hydrolysis (carboxylic acid) + hydroxy (isomer 2)	-	-	-	-	+	-	+	-	-	+	+	-	+
CM25	ester hydrolysis (carboxylic acid) + hydroxy (isomer 3)	+	-	-	-	+	+	+	-	-	+	+	-	+
CM26	ester hydrolysis (carboxylic acid) + dihydroxy (isomer 1)	-	-	-	-	-	-	-	-	-	+	+	-	-
CM27	ester hydrolysis (carboxylic acid) + dihydroxy (isomer 2)	-	-	-	-	-	-	-	-	-	+	+	-	+
CM28	ester hydrolysis (carboxylic acid) + dihydroxy (isomer 3)	+	-	-	-	-	-	-	-	-	+	+	-	+
CM29	ester hydrolysis (carboxylic acid) + gluc	+	-	-	-	-	-	-	-	-	-	-	-	-
CM30	ester hydrolysis (8-hydroxyquinolin)	+	+	+	+	+	+	+	+	+	+	+	+	+
CM31	ester hydrolysis (8-hydroxyquinolin) + hydroxy	-	+	+	+	+	+	+	+	+	+	+	+	-
CM32	ester hydrolysis (8-hydroxyquinolin) + sulfate	+	-	-	-	-	-	-	-	-	-	-	-	-
CM33	ester hydrolysis (8-hydroxyquinolin) + gluc	+	-	-	-	-	-	-	-	-	-	-	-	-
CM34	ester hydrolysis (8-hydroxyquinolin) + hydroxy + gluc	+	-	-	-	-	-	-	-	-	-	-	-	-

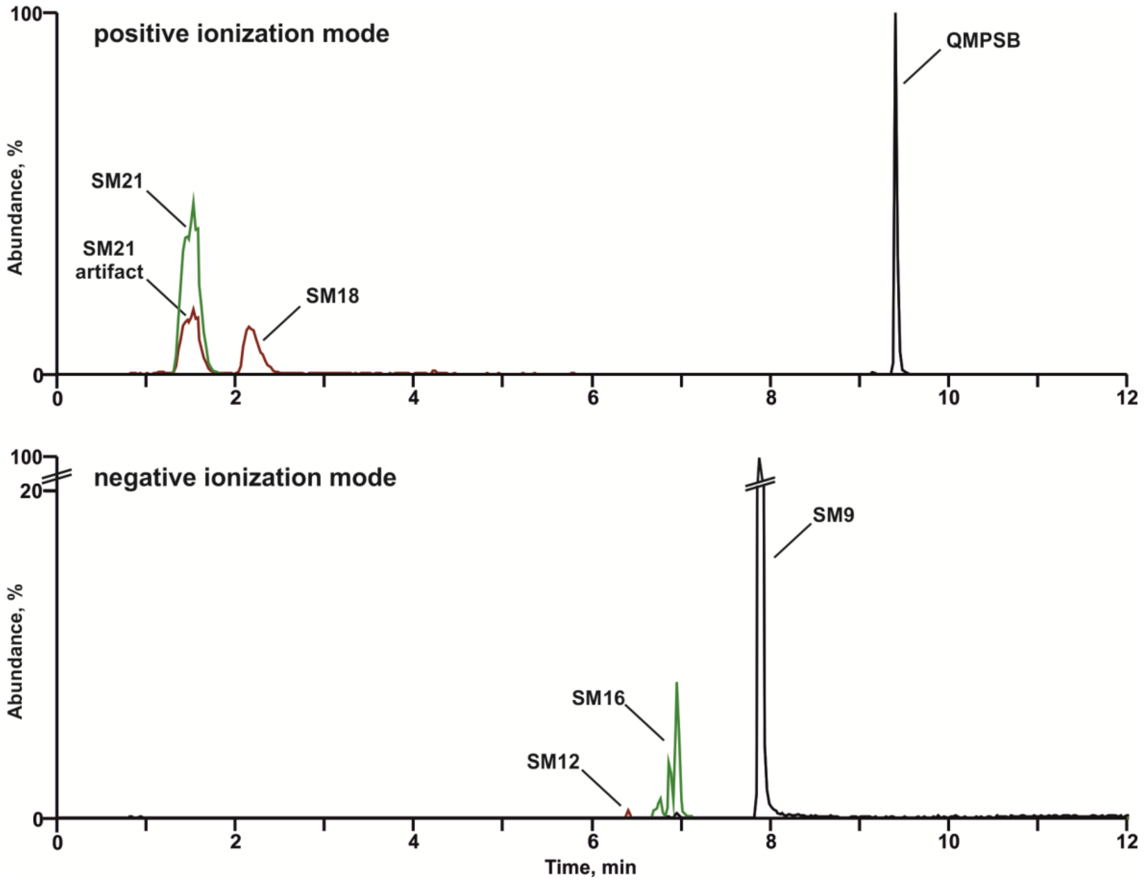

Figure S1. Chromatograms of QMPSB and its five most abundant metabolites in pHLS9 incubation (6h sample) in positive or negative ionization mode: QMPSB [*m/z* 411.1373], SM9 [ester hydrolysis (carboxylic acid), *m/z* 282.0806], SM12 [ester hydrolysis (carboxylic acid) + hydroxy (isomer 1), *m/z* 298.0755], SM16 [ester hydrolysis (carboxylic acid) + glucuronic acid, *m/z* 458.1126], SM18 [ester hydrolysis (8-hydroxyquinoline), *m/z* 146.0600], SM21 [ester hydrolysis (8-hydroxyquinoline) + glucuronic acid, *m/z* 322.0921]; SM21 artifact [deglucuronidated in ESI source, *m/z* 146.0600].

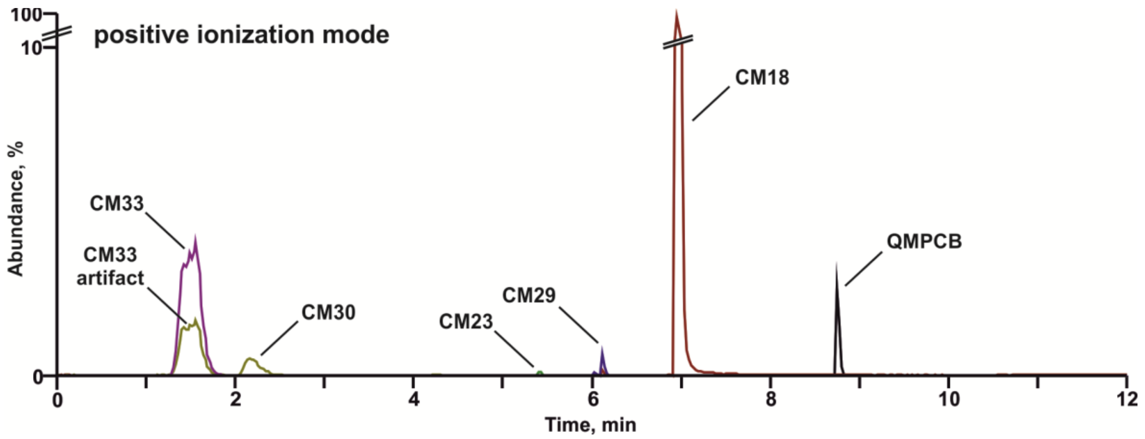

Figure S2. Chromatograms of QMPCB and its five most abundant metabolites in pHLS9 incubation (6h sample) in positive ionization mode: QMPCB [*m/z* 375.1703], CM18 [ester hydrolysis (carboxylic acid), *m/z* 248.1281], CM23 [ester hydrolysis (carboxylic acid) + hydroxy (isomer 1), *m/z* 264.1230], CM29 [ester hydrolysis (carboxylic acid) + glucuronic acid, *m/z* 424.1602], CM30 [ester hydrolysis (8-hydroxyquinoline), *m/z* 146.0600], CM33 [ester hydrolysis (8-hydroxyquinoline) + glucuronic acid, *m/z* 322.0921]; CM33 artifact [deglucuronidated in ESI source, *m/z* 146.0600].

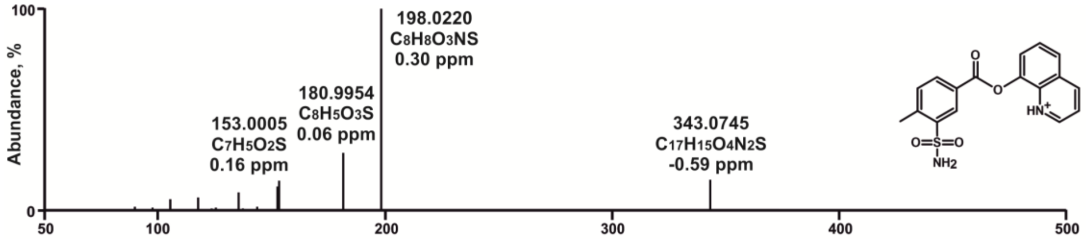

MS² of SM1 (N,N-bisdealkyl, *m/z* at 343.0747, RT 7.6 min, pos)

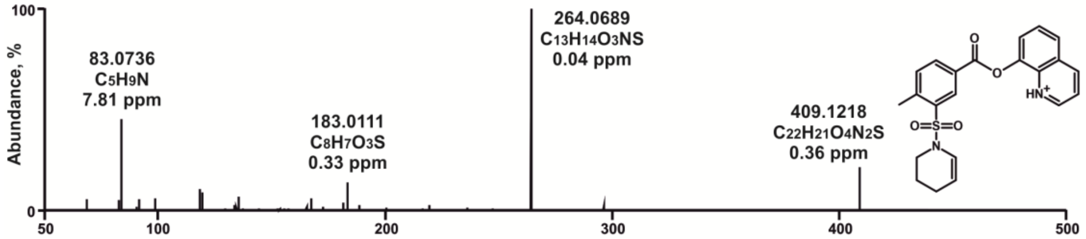

MS² of SM2 (dehydro (isomer 1), *m/z* at 409.1217, RT 8.8 min, pos)

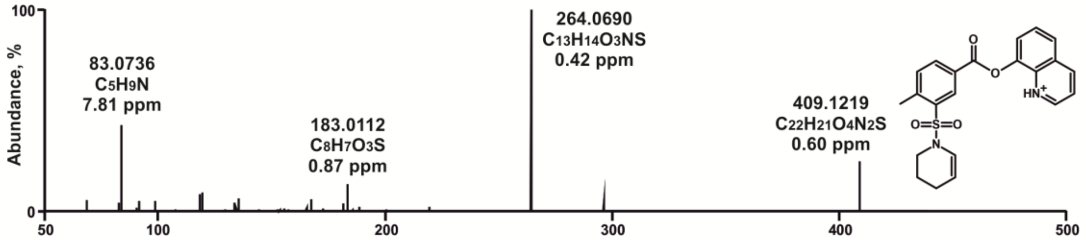

MS² of SM3 (dehydro (isomer 2), *m/z* at 409.1217, RT 9.5 min, pos)

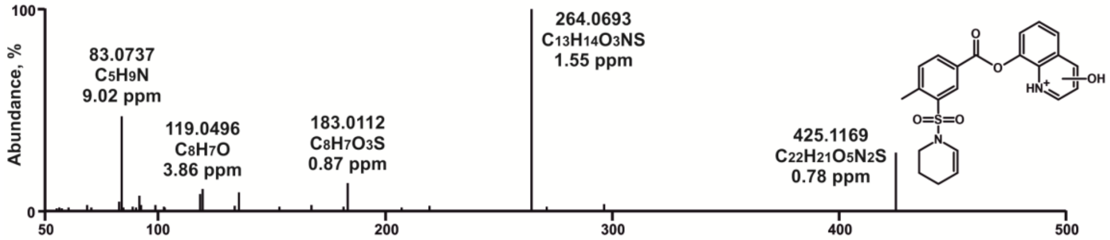

MS² of SM4 (hydroxy + dehydro, *m/z* at 425.1166, RT 8.1 min, pos)

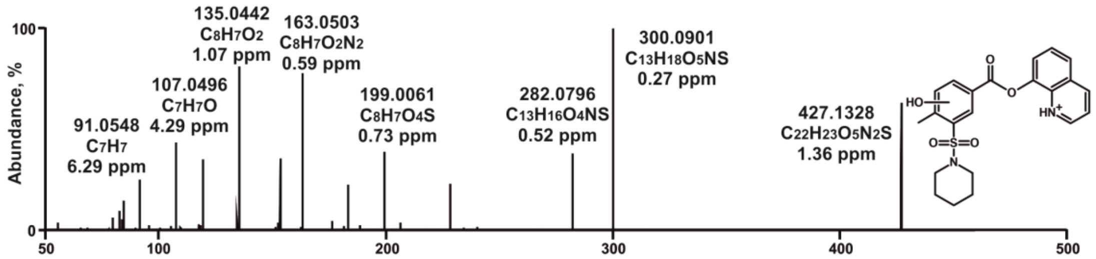

MS² of SM5 (hydroxy (isomer 1), *m/z* at 427.1322, RT 8.2 min, pos)

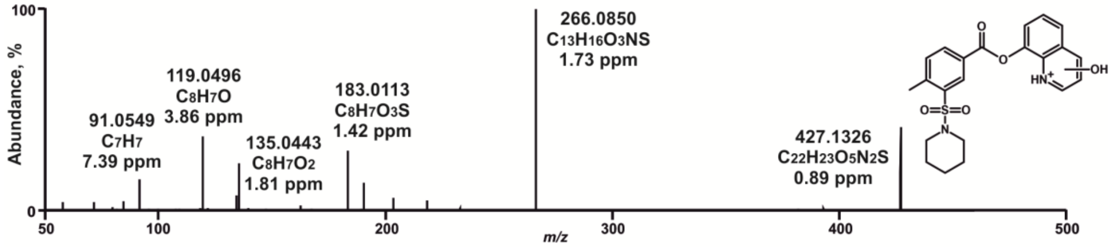

MS² of SM6 (hydroxy (isomer 2), *m/z* at 427.1322, RT 8.8 min, pos)

Figure S3. High-resolution MS² spectra of QMPSB metabolites detected in pHLS9 and monooxygenases activity screening. RT, retention time; pos, positive ionization mode; neg, negative ionization mode; gluc, glucuronic acid.

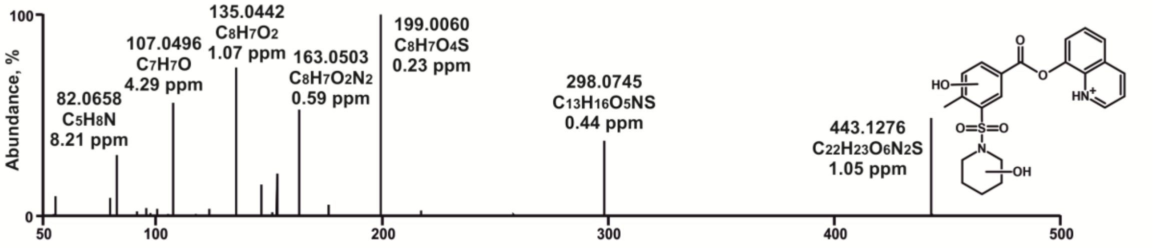

MS^2 of SM7 (dihydroxy (isomer 1), *m/z* at 443.1271, RT 8.1 min, pos)

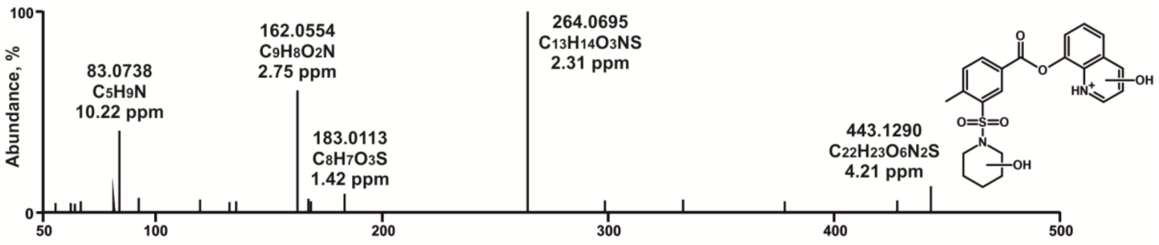

MS^2 of SM8 (dihydroxy (isomer 2), *m/z* at 443.1271, RT 8.1 min, pos)

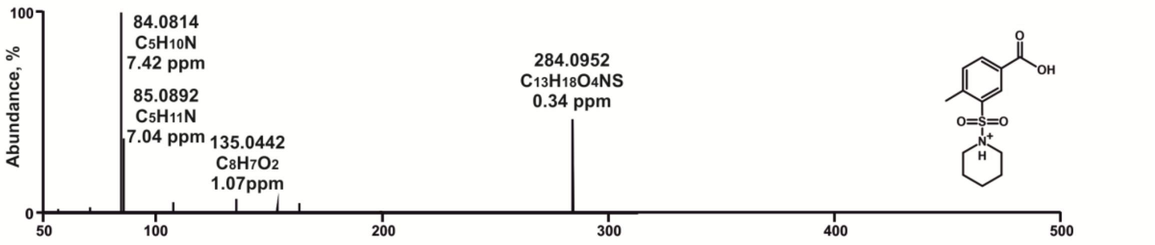

MS^2 of SM9 (ester hydrolysis (carboxylic acid), *m/z* at 284.0951, RT 7.9 min, pos)

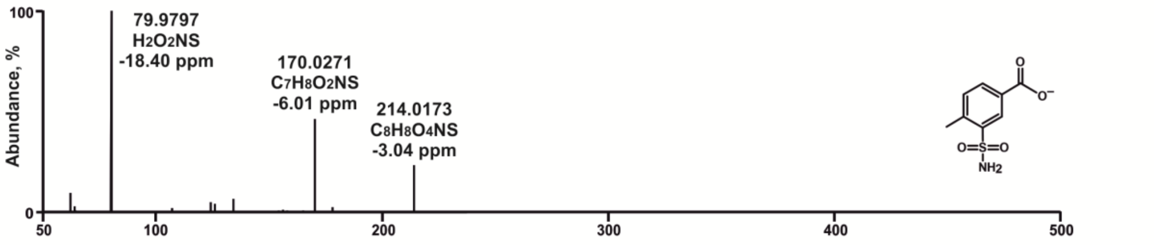

MS^2 of SM10 (ester hydrolysis (carboxylic acid) + N,N-bisdealkyl, *m/z* at 214.0180, RT 5.0 min, neg)

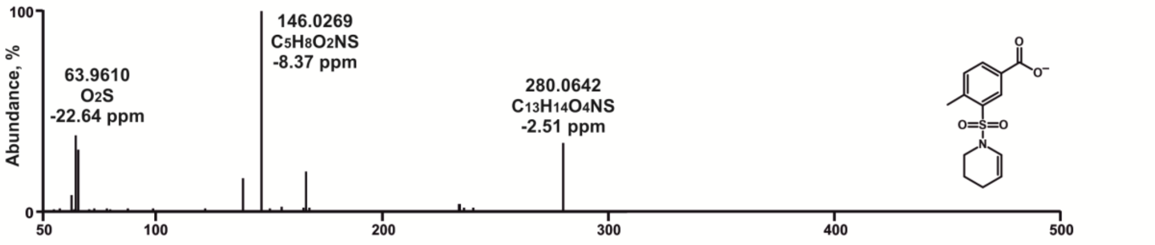

MS^2 of SM11 (ester hydrolysis (carboxylic acid) + dehydro, *m/z* at 280.0649, RT 8.0 min, neg)

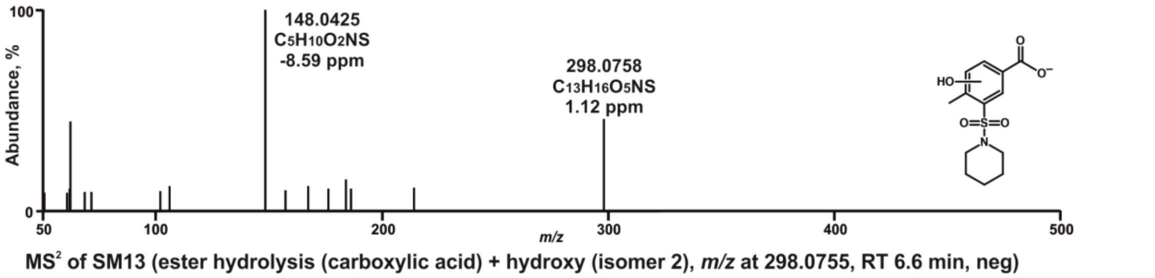

MS^2 of SM13 (ester hydrolysis (carboxylic acid) + hydroxy (isomer 2), *m/z* at 298.0755, RT 6.6 min, neg)

Figure S3. (continued)

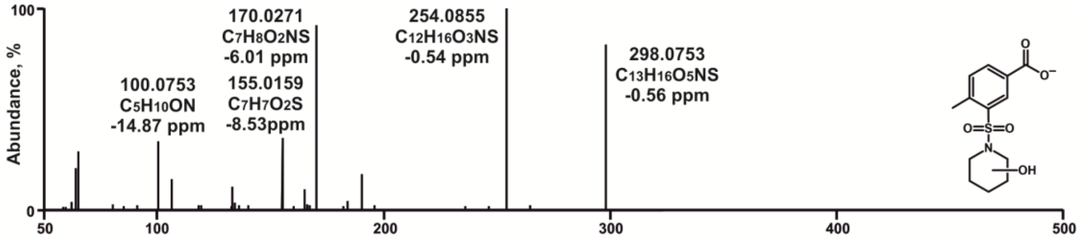

MS^2 of SM14 (ester hydrolysis (carboxylic acid) + hydroxy (isomer 3), *m/z* at 298.0755, RT 7.0 min, neg)

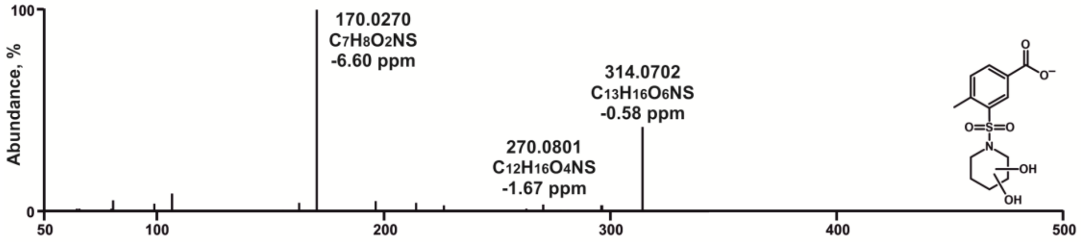

MS^2 of SM15 (ester hydrolysis (carboxylic acid) + dihydroxy, *m/z* at 314.0704, RT 6.2 min, neg)

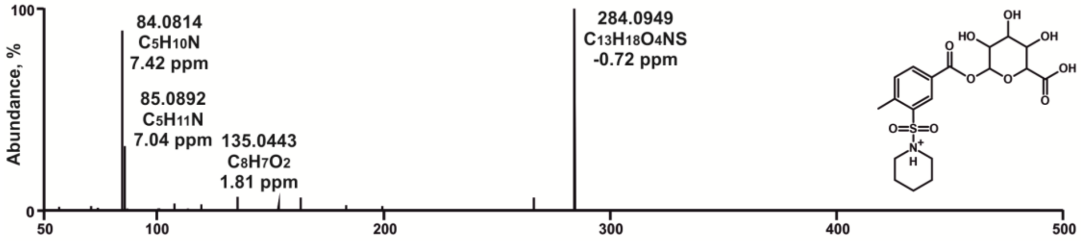

MS^2 of SM16 (ester hydrolysis (carboxylic acid) + gluc, *m/z* at 460.1272, RT 6.9 min, pos)

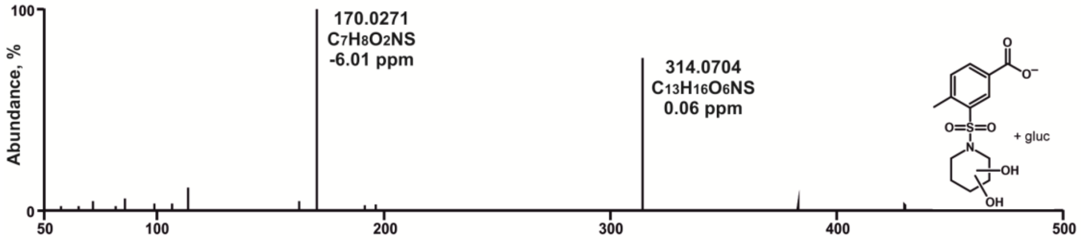

MS^2 of SM17 (ester hydrolysis (carboxylic acid) + dihydroxy + gluc, *m/z* at 490.1025, RT 5.6 min, neg)

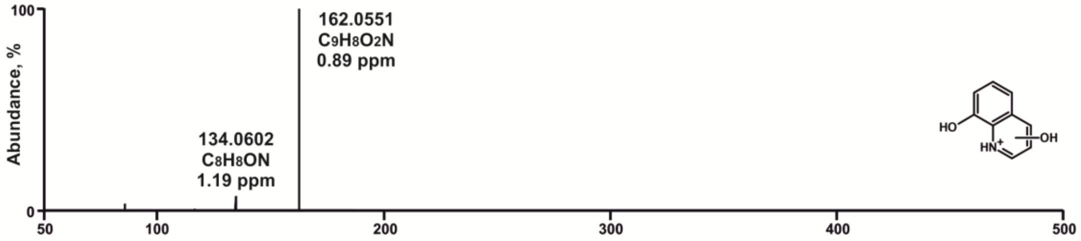

MS^2 of SM19 (ester hydrolysis (8-hydroxyquinoline) + hydroxy, *m/z* at 162.0550, RT 1.3 min, pos)

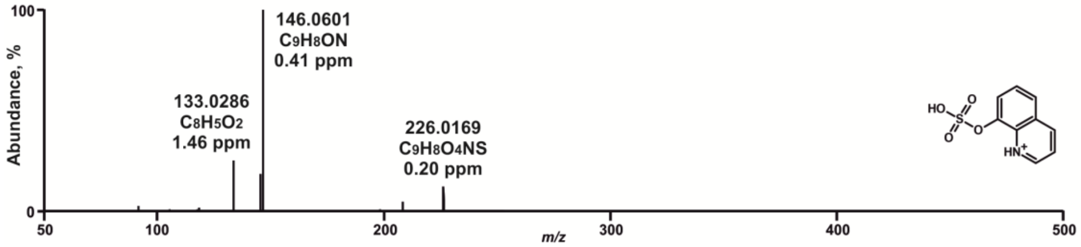

MS^2 of SM20 (ester hydrolysis (8-hydroxyquinoline) + sulfate, *m/z* at 226.0169, RT 2.2 min, pos)

Figure S3. (continued)

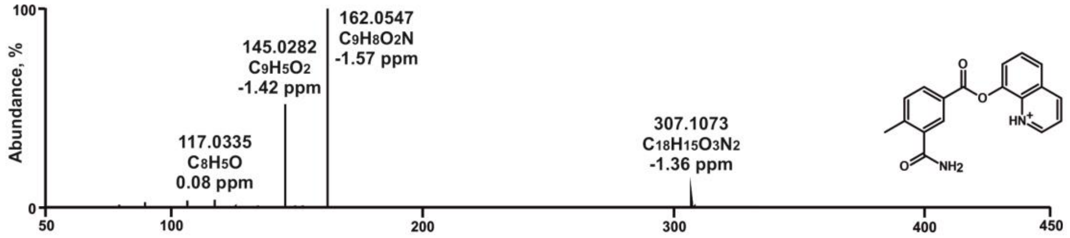

MS^2 of CM1 (N,N-bisdealkyl, *m/z* at 307.1077, RT 7.2 min, pos)

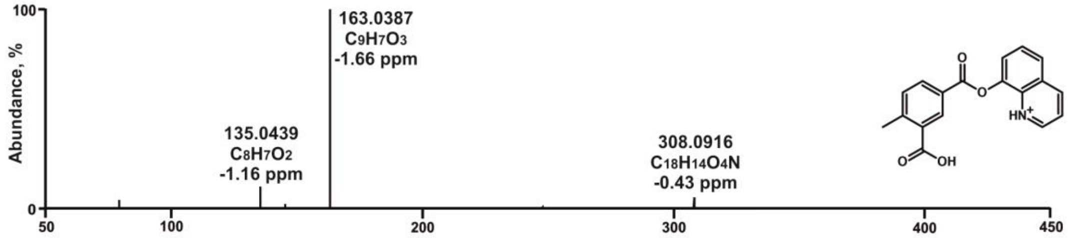

MS^2 of CM2 (carboxamide cleavage (carboxylic acid), *m/z* at 308.0917, RT 8.0 min, pos)

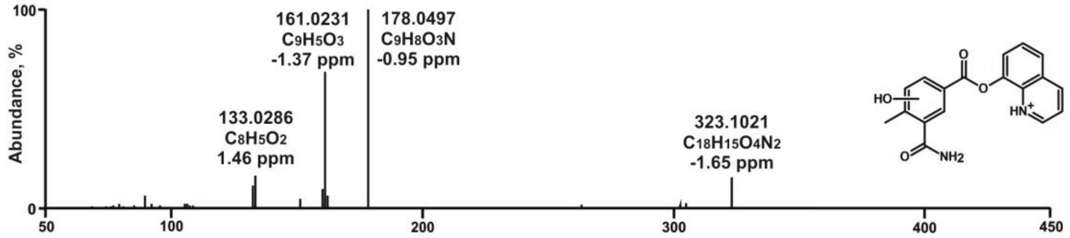

MS^2 of CM3 (N,N-bisdealkyl + hydroxy, *m/z* at 323.1026, RT 6.5 min, pos)

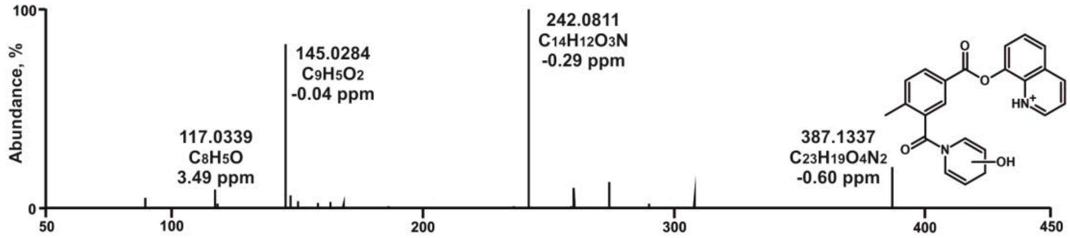

MS^2 of CM4 (hydroxy + didehydro, *m/z* at 387.1339, RT 8.1 min, pos)

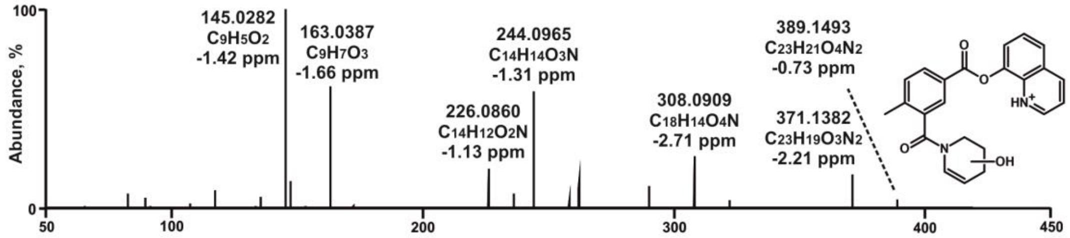

MS^2 of CM5 (hydroxy + dehydro (isomer 1), *m/z* at 389.1496, RT 7.1 min, pos)

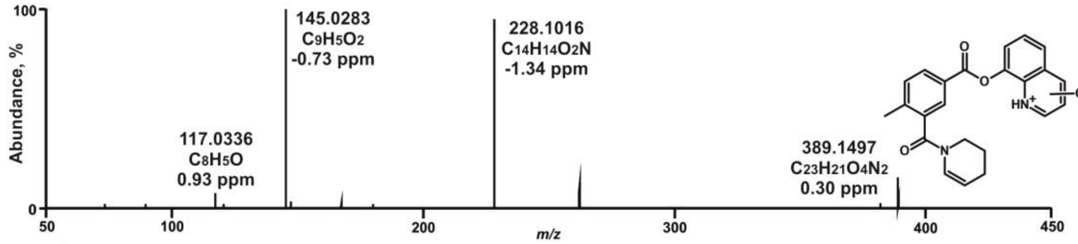

MS^2 of CM6 (hydroxy + dehydro (isomer 2), *m/z* at 389.1496, RT 7.4 min, pos)

Figure S4. High-resolution MS^2 spectra of QMPCB metabolites detected in pHLS9 and monooxygenases activity screening. RT, retention time; pos, positive ionziation mode; neg, negative ionization mode; gluc, glucuronic acid.

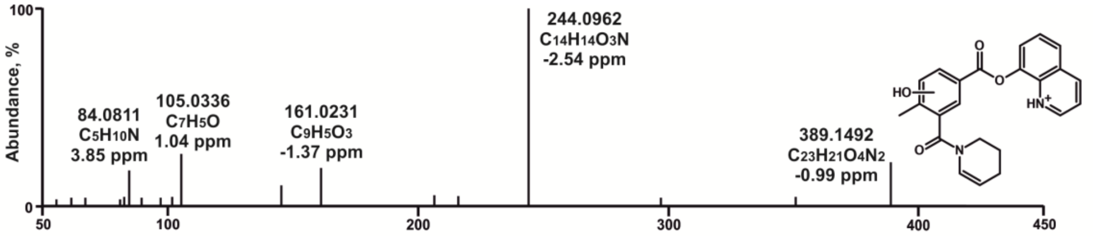

MS^2 of CM7 (hydroxy + dehydro (isomer 3), *m/z* at 389.1496, RT 8.6 min, pos)

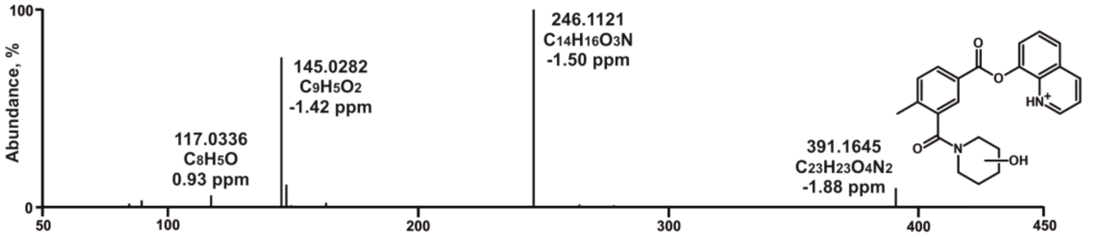

MS^2 of CM8 (hydroxy (isomer 1), *m/z* at 391.1652, RT 7.3 min, pos)

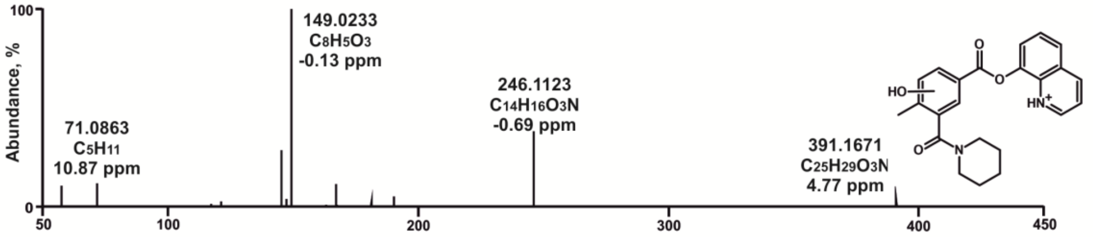

MS^2 of CM9 (hydroxy (isomer 2), *m/z* at 391.1652, RT 7.3 min, pos)

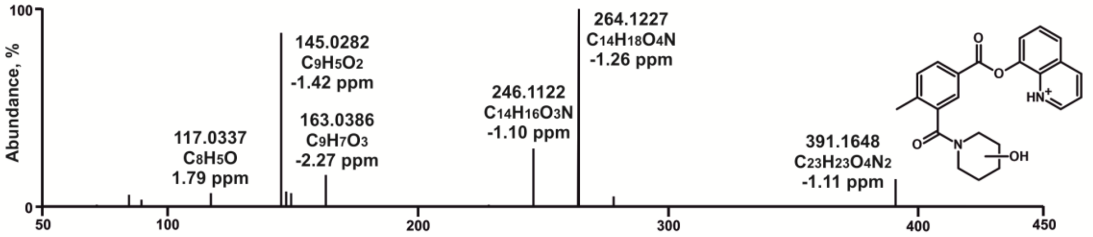

MS^2 of CM10 (hydroxy (isomer 3), *m/z* at 391.1652, RT 7.6 min, pos)

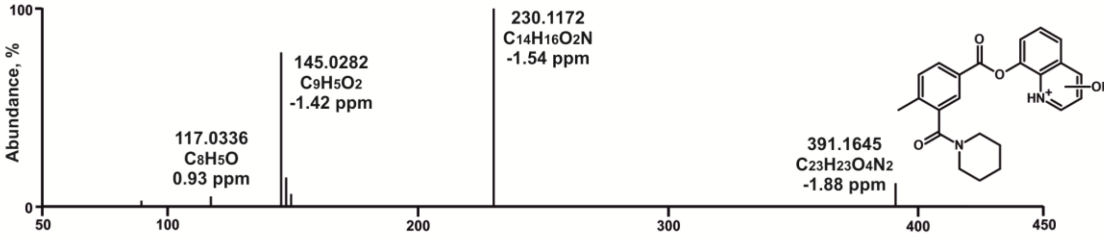

MS^2 of CM11 (hydroxy (isomer 4), *m/z* at 391.1652, RT 8.0 min, pos)

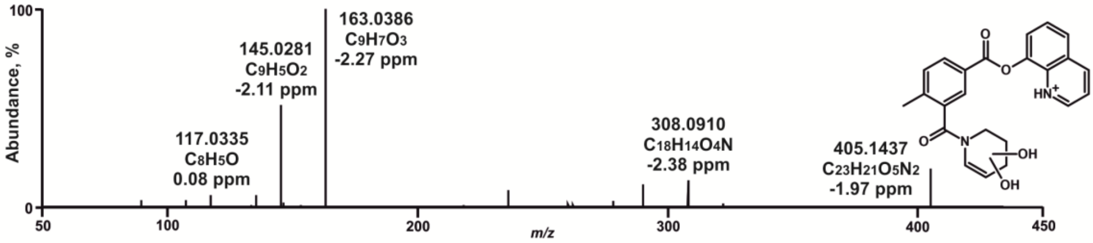

MS^2 of CM12 (dihydroxy + dehydro (isomer 1), *m/z* at 405.1445, RT 7.2 min, pos)

Figure S4. (continued)

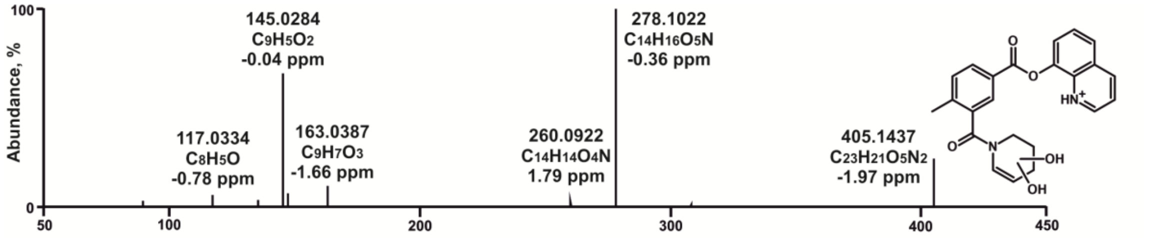

MS² of CM13 (dihydroxy + dehydro (isomer 2), *m/z* at 405.1445, RT 7.6 min, pos)

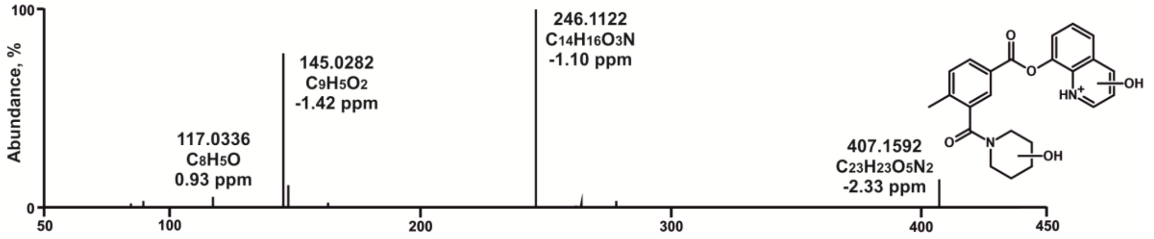

MS² of CM14 (dihydroxy (isomer 1), *m/z* at 407.1601, RT 6.6 min, pos)

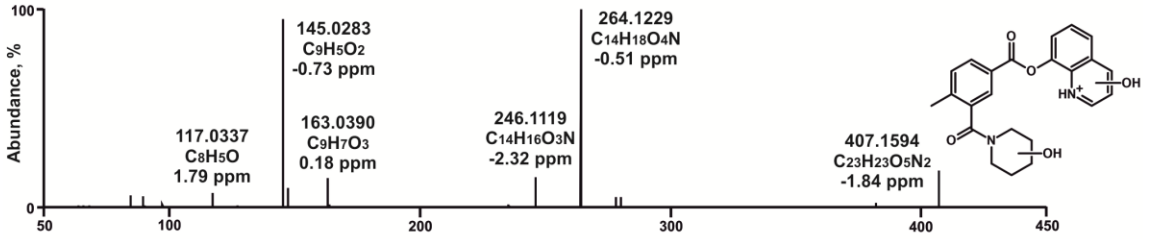

MS² of CM15 (dihydroxy (isomer 2), *m/z* at 407.1601, RT 6.9 min, pos)

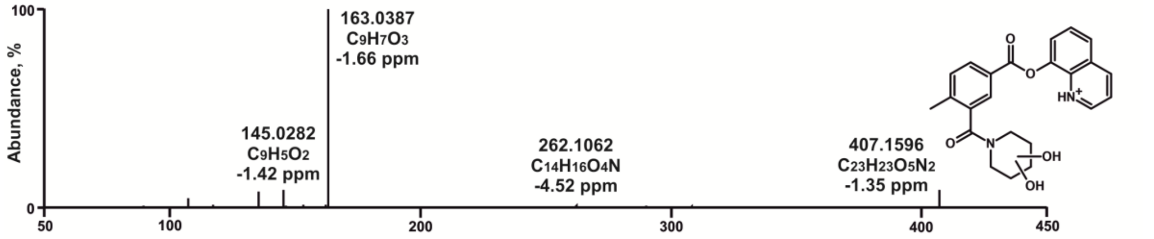

MS² of CM16 (dihydroxy (isomer 3), *m/z* at 407.1601, RT 7.6 min, pos)

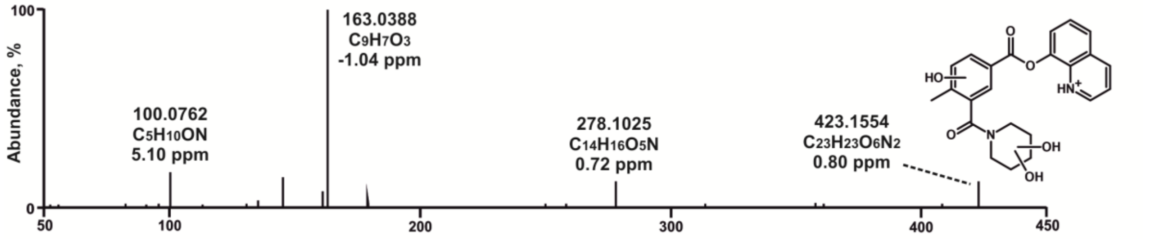

MS² of CM17 (trihydroxy, *m/z* at 423.1551, RT 7.1 min, pos)

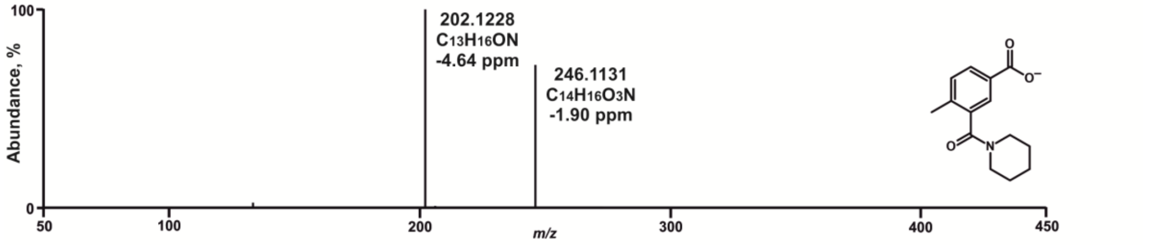

MS² of CM18 (ester hydrolysis (carboxylic acid), *m/z* at 246.1136, RT 7.0 min, neg)

Figure S4. (continued)

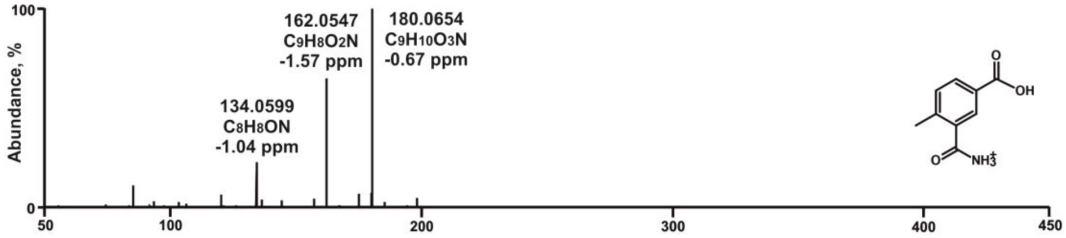

MS^2 of CM19 (ester hydrolysis (carboxylic acid) + N,N-bisdealkyl, *m/z* at 180.0655, RT 0.6 min, pos)

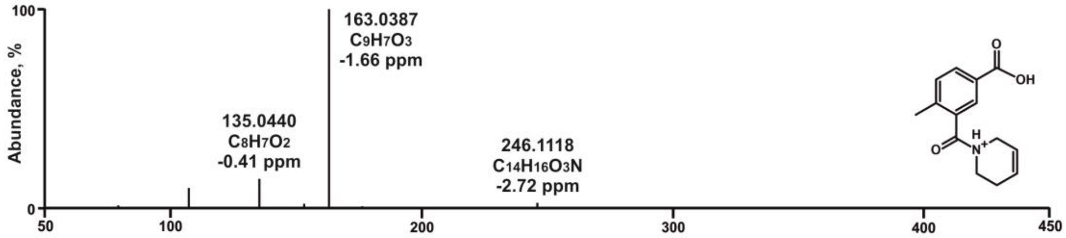

MS^2 of CM20 (ester hydrolysis (carboxylic acid) + dehydro (isomer 1), *m/z* at 246.1125, RT 6.3 min, pos)

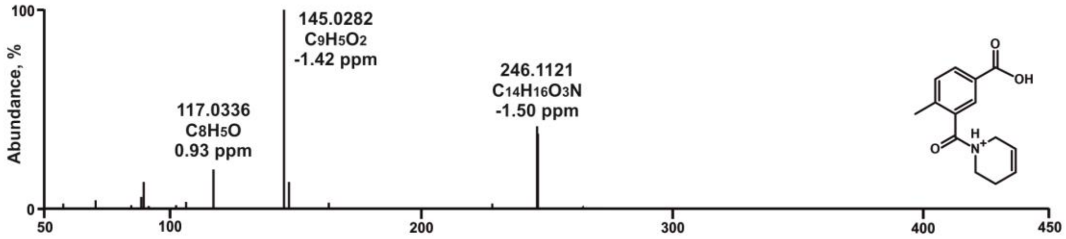

MS^2 of CM21 (ester hydrolysis (carboxylic acid) + dehydro (isomer 2), *m/z* at 246.1125, RT 7.3 min, pos)

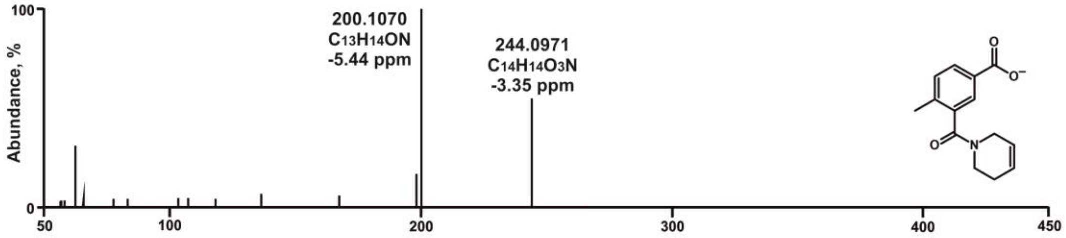

MS^2 of CM21 (ester hydrolysis (carboxylic acid) + dehydro (isomer 2), *m/z* at 244.0979, RT 7.3 min, neg)

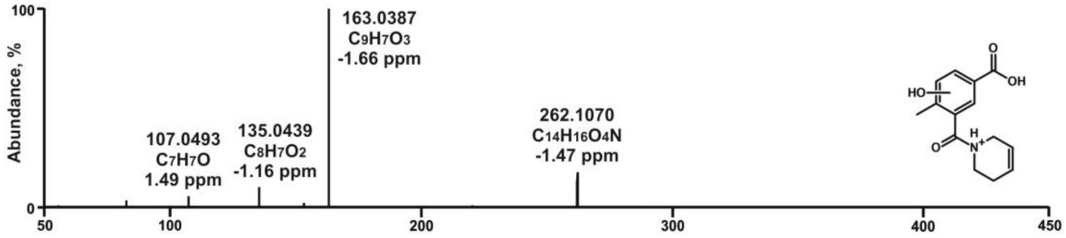

MS^2 of CM22 (ester hydrolysis (carboxylic acid) + hydroxy + dehydro, *m/z* at 262.1074, RT 5.4 min, pos)

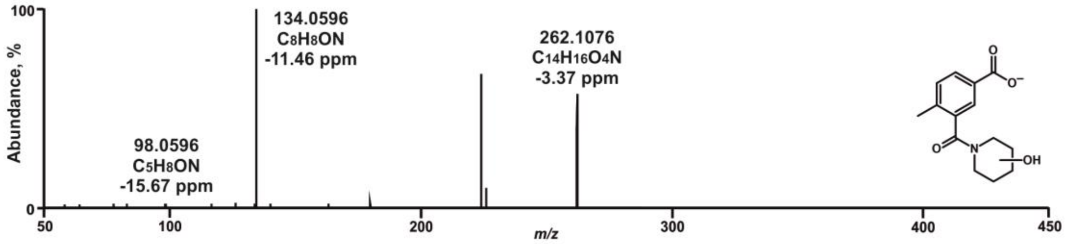

MS^2 of CM23 (ester hydrolysis (carboxylic acid) + hydroxy (isomer1), *m/z* at 262.1085, RT 5.4 min, neg)

Figure S4. (continued)

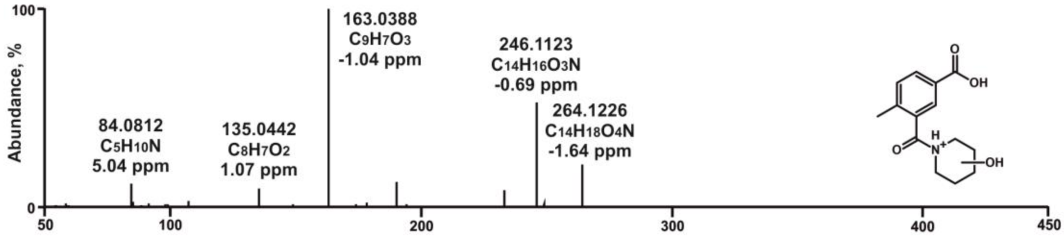

MS^2 of CM24 (ester hydrolysis (carboxylic acid) + hydroxy (isomer 2), *m/z* at 264.1230, RT 5.7 min, pos)

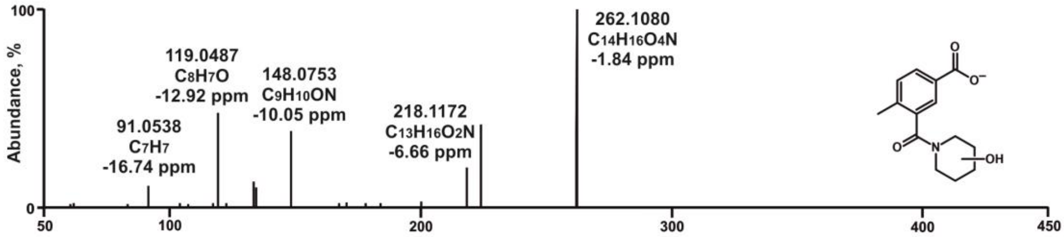

MS^2 of CM24 (ester hydrolysis (carboxylic acid) + hydroxy (isomer 2), *m/z* at 262.1085, RT 5.7 min, neg)

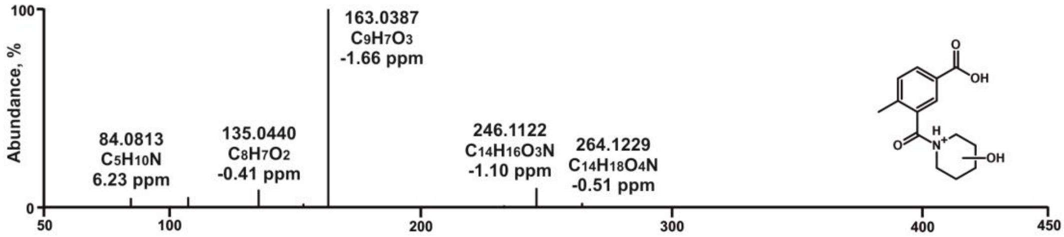

MS^2 of CM25 (ester hydroylsis (carboxylic acid) + hydroxy (isomer 3), *m/z* at 264.1230, RT 6.3 min, pos)

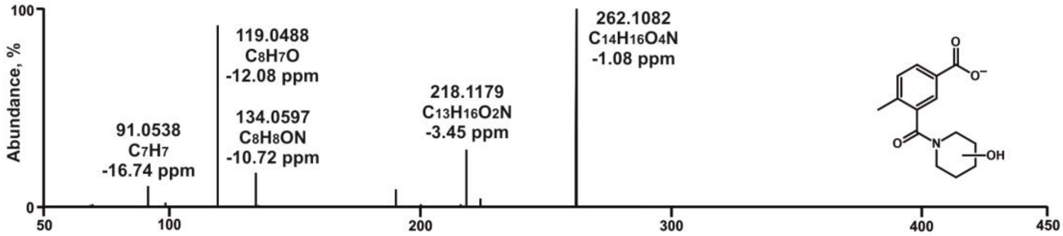

MS^2 of CM25 (ester hydrolysis (carboxylic acid) + hydroxy (isomer 3), *m/z* at 262.1085, RT 6.3 min, neg)

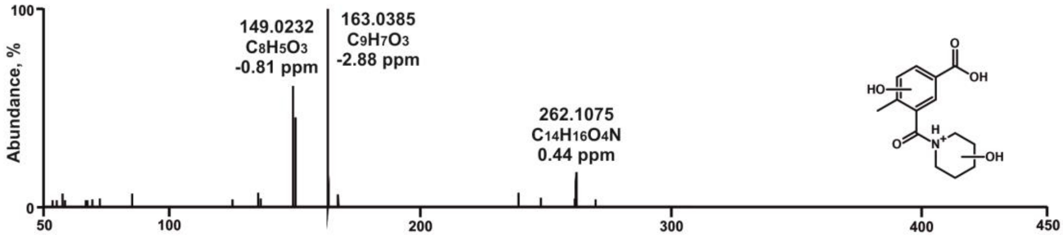

MS^2 of CM26 (ester hydrolysis (carboxylic acid) + dihydroxy (isomer 1), *m/z* at 280.1179, RT 5.0 min, pos)

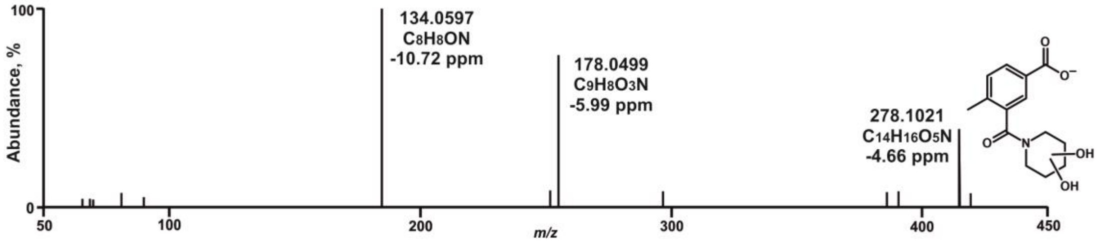

MS^2 of CM27 (ester hydrolysis (carboxylic acid) + dihydroxy (isomer 2), *m/z* at 278.1034, RT 5.0 min, neg)

Figure S4. (continued)

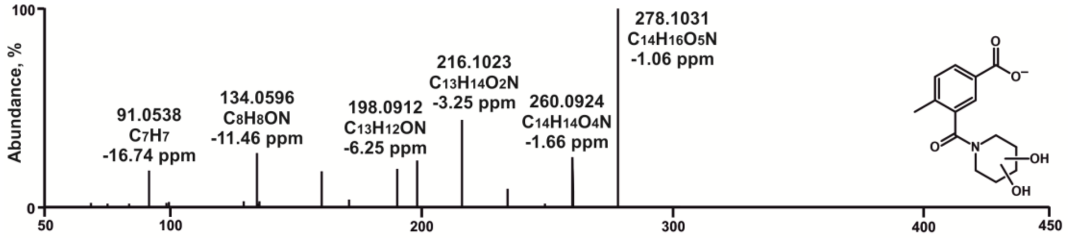

MS^2 of CM28 (ester hydrolysis (carboxylic acid) + dihydroxy (isomer 3), *m/z* at 278.1034, RT 5.7 min, neg)

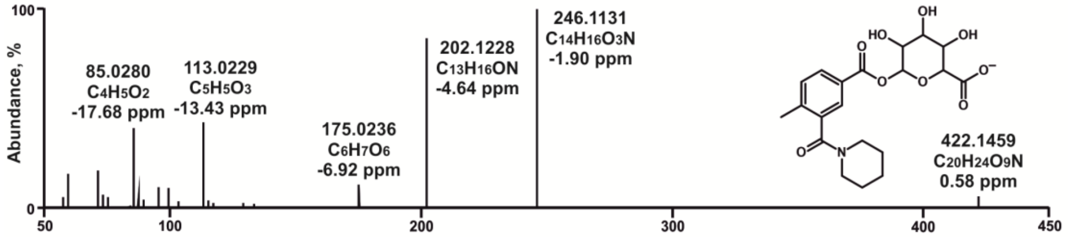

MS^2 of CM29 (ester hydrolysis (carboxylic acid) + gluc, *m/z* at 422.1457, RT 6.1 min, neg)

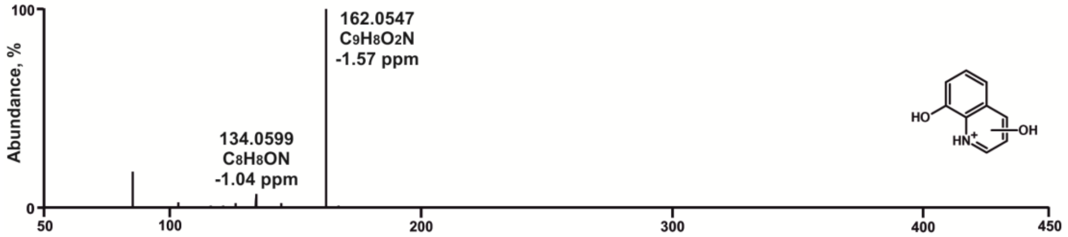

MS^2 of CM31 (ester hydrolysis (8-hydroxyquinoline) + hydroxy, *m/z* at 162.0550, RT 1.2 min, pos)

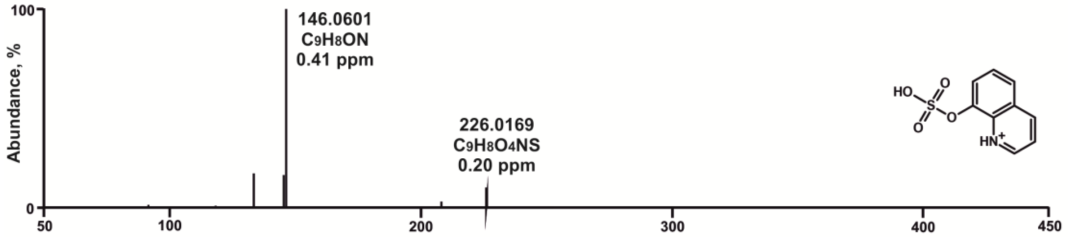

MS^2 of CM32 (ester hydrolysis (8-hydroxyquinoline) + sulfate, *m/z* at 226.0169, RT 2.2 min, pos)

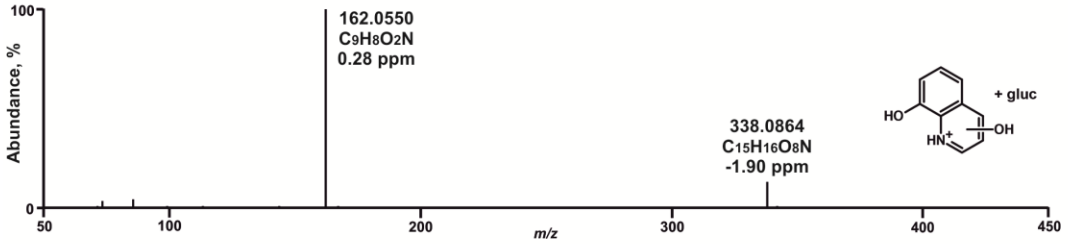

MS^2 of CM34 (ester hydrolysis (8-hydroxyquinoline) + hydroxy + gluc, *m/z* at 338.0870, RT 0.90 min, pos)

Figure S4. (continued)
